# U-FISH: a fluorescent spot detector for imaging-based spatial-omics analysis and AI-assisted FISH diagnosis

**DOI:** 10.1186/s13059-025-03736-x

**Published:** 2025-09-01

**Authors:** Weize Xu, Huaiyuan Cai, Qian Zhang, Zhengze Wang, Jiajun Yang, Xiaofeng Wu, Chengwen Li, Chenghua Cui, Changzhi Liu, Jin He, Florian Mueller, Jinxia Dai, Chen Hao, Wei Ouyang, Gang Cao

**Affiliations:** 1 Faculty of Life and Health Sciences, Shenzhen University of Advanced Technology, 518107 Shenzhen, China; 2 College of Veterinary Medicine, Huazhong Agricultural University, Wuhan, China; 3 State Key Laboratory of Agricultural Microbiology, Huazhong Agricultural University, Wuhan, China; 4 College of Informatics, Huazhong Agricultural University, Wuhan, China; 5 College of Life Science and Technology, Huazhong Agricultural University, Wuhan, China; 6 The Brain Cognition and Brain Disease Institute, Shenzhen Institutes of Advanced Technology, Chinese Academy of Sciences, 518055 Shenzhen, China; 7 Institute of Hematology & Blood Diseases Hospital, Chinese Academy of Medical Sciences & Peking Union Medical College, Tianjin, China; 8 SpatialFISH Biotechnology Company Limited, Shenzhen, China; 9 Institut Pasteur, Université Paris Cité, Centre de Ressources et Recherches Technologiques (UTechS-PBI, C2RT), F-75015, Paris, France; 10 Department of Applied Physics, Science for Life Laboratory, KTH Royal Institute of Technology, Stockholm, Sweden; 11 Department of Computer Science and Engineering, Hong Kong University of Science and Technology, Hong Kong, China; 12 Current address: Department of Genetics, Stanford University School of Medicine, Stanford, CA USA

## Abstract

Imaging-based spatial-omics advances biomedical discoveries with subcellular resolution and high sensitivity, but accurately identifying signal spots from diverse images remains challenging. We develop U-FISH, a deep learning method that enhances images for consistent spot detection across various spatial-omics data. We establish a comprehensive FISH image dataset from seven spatial-omics methods. Benchmark analysis shows U-FISH has superior accuracy and generalizability compared to existing methods and can effectively decode 3D FISH data. U-FISH is the first spot detection software integrated with large language models, as demonstrated in AI-assisted diagnostics. Our study provides a valuable tool for spatial-omics analysis and diagnostics.

**Supplementary Information:**

The online version contains supplementary material available at 10.1186/s13059-025-03736-x.

## Background

Imaging technologies play a pivotal role in biomedical research. In the study of gene regulation, methods with single-molecule sensitivity have provided invaluable insight into our understanding of how individual cells regulate their genome in space and time [1]. Fluorescent in situ hybridization (FISH) techniques are instrumental approaches permitting the visualization of individual RNAs or genomic loci with high spatial resolution in their native cellular environment. Initial implementations of single-molecule FISH (smFISH) are used to probe a handful of different mRNA species [2, 3]. Additionally, DNA FISH plays a significant role in diagnosing and researching genetic diseases, helping identify abnormal gene locations and structural changes [4, 5]. Recent advancements in probe synthesis, multiplexed barcoded detections, signal amplification and tissue clearing, opened the door for the field of spatial transcriptomics and spatial genomics. These approaches now enable the visualization of hundreds to thousands of different transcripts or genetic loci with single-molecule sensitivity in complex tissues at the 3D level [6–11]. However, the analysis of these increasingly large and complex datasets remains a challenge, particularly, the accurate and efficient detection of diffraction-limited RNA spots, which is incredibly challenging in images with varying background levels and spot intensities. Many rule-based methods have been developed to address the challenge of spot detection in microscope images [12–16]. However, a significant limitation of these methods lies in their inability to uniformly apply across different datasets without parameter adjustments. This limitation stems from the varying imaging conditions and sample characteristics across datasets, which introduce distinct features requiring time-consuming parameter tuning for each image.

In the evolving field of biomedical imaging, deep learning has revolutionized both traditional methods and introduced novel approaches to image analysis. Technologies such as Cellpose [17, 18] for cell segmentation, CARE [19, 20] for image reconstruction, and advanced techniques for cell image classification [21] stand as pillars of this transformation. Each of these applications demonstrates deep learning’s capacity to simplify and significantly enhance the precision and efficiency of analyzing microscopic images. Building upon these foundational achievements, deep learning has extended its influence to the specific challenges of spot detection in microscopy images. This advancement is crucial for understanding cellular behaviors and gene expression patterns at a granular level. Techniques such as DetNet [22], SpotLearn [23], and deepBlink [24] leverage deep learning models to achieve FISH spot detection without the need for manual parameter tuning. This automation represents a significant advancement over traditional methods, streamlining the detection process and enhancing accuracy. Additionally, DeepSpot [25] specializes in enhancing RNA spots in single-molecule FISH (smFISH) microscopy images. However, when it comes to applying these methods in real-world scenarios, notable shortcomings remain to be addressed. First and foremost, there is a lack of sufficiently broad datasets, which hampers the training of versatile models capable of effectively dealing with FISH data from diverse sources. Additionally, these methods still exhibit gaps in their ability to process 3D data and usability, making the transition from theoretical application to practical use somewhat challenging.

**Fig. 1:**
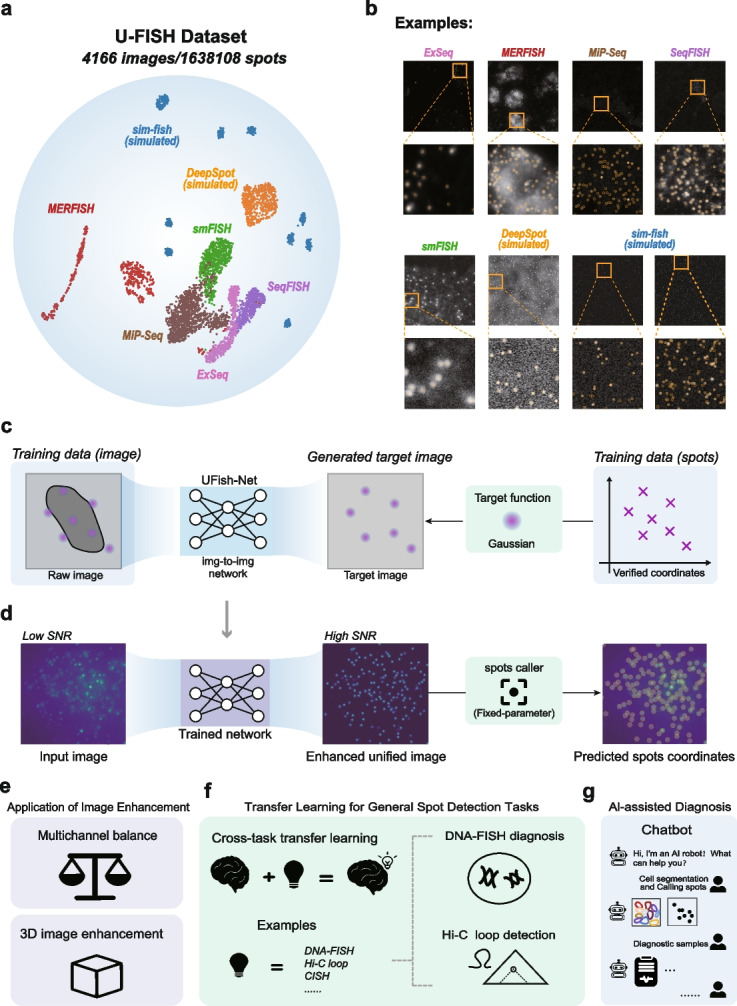
U-FISH dataset and method. **a** Scatter plot illustrating the UMAP dimensionality reduction of the images in the U-FISH dataset. This diverse dataset encompasses a total of 4166 images sourced from seven different origins, featuring 1,638,108 manually verified spots. UMAP visualization captures the dataset’s inherent diversity, showcasing the variance in data distribution and complexity across different sources. **b** Sample images from each of the seven distinct sources, accompanied by annotations of spot coordinates, are displayed. These examples highlight the variability in background characteristics and signal spot features among the different sources. This diversity underscores the comprehensive nature of the U-FISH dataset, ensuring its robustness and applicability across a wide range of FISH imaging scenarios. **c** During training, the model leverages coordinate information from annotations to generate the target images using gaussian function. The img-to-img model is then trained using these target images alongside the original images to learn the transformation. **d** During the inference phase, the trained model inputs original images, typically characterized by low signal-to-noise ratio (SNR), and outputs enhanced images with consistent features and significantly reduced background noise, resulting in a higher SNR. Spot detection is then executed on these enhanced images using a fixed-parameter, rule-based algorithm (see the “Methods” section). **e**–**g** Downstream applications of U-FISH pre-trained models and enhanced images

To address the need for a universal model in imaging-based spatial omics and clinical diagnosis, we propose U-FISH, a deep learning method for spot detection. We leveraged a comprehensive dataset of over 4000 images and 1.6 million signal spots from seven sources (Fig. 1a, b) to train a U-Net model that transforms diverse raw images into enhanced, uniform images for reliable spot detection with fixed parameters (Fig. 1c, d). U-FISH demonstrates superior accuracy and generalizability over existing methods and introduces an innovative approach enabling a 2D network to effectively process 3D FISH data (Fig. 1e), overcoming a common limitation. Furthermore, this compact (163k parameters) and robust foundational model can be easily fine-tuned for a wide range of applications beyond RNA detection, including DNA-FISH diagnostics, Hi-C loop identification, and CISH signal analysis (Fig. 1f). To ensure broad accessibility, we developed intuitive GUI and Chatbot plugins, making U-FISH the first spot detection software integrated with large language models (LLMs) to simplify use for all researchers (Fig. 1g).

## Results

### U-FISH enabling universal, rapid, and precise detection of FISH spots

To ensure consistent detection performance across different FISH datasets without the need for manual parameter adjustments, U-FISH employs a U-Net model [26] to transform raw FISH images with variable characteristics into enhanced images with uniform signal spot characteristics and an improved signal-to-noise ratio. This approach allows for efficient signal spot detection without time-consuming manual parameter tuning. A cornerstone feature of our approach is the U-FISH dataset, a meticulously curated collection comprising over 4000 images with more than 1.6 million verified targets from seven diverse sources. This data diversity allows us to train a universal spot detection model and conduct an unbiased evaluation of the algorithm’s performance (Fig. 1a–d, Additional file 1: Fig. S1). Detailed information on the dataset and model specifics are provided in the “Methods” section.

In addition to its spot detection capabilities in RNA-FISH images, U-FISH also integrates several other functionalities. The robust network architecture underlying U-FISH can be easily adapted to similar tasks, such as loop detection in Hi-C data and clinical diagnostics through DNA-FISH signal recognition. The enhance function of U-FISH effectively balances fluorescence intensity across channels and enables precise 3D signal spot detection from enhanced images. Moreover, the integration with chatbot interfaces allows U-FISH to facilitate image recognition through human-machine dialogue. The combination of these features positions U-FISH as a powerful and flexible tool for various biomedical imaging applications, further promoting its adoption in research and clinical settings (Fig. 1e).

**Fig. 2:**
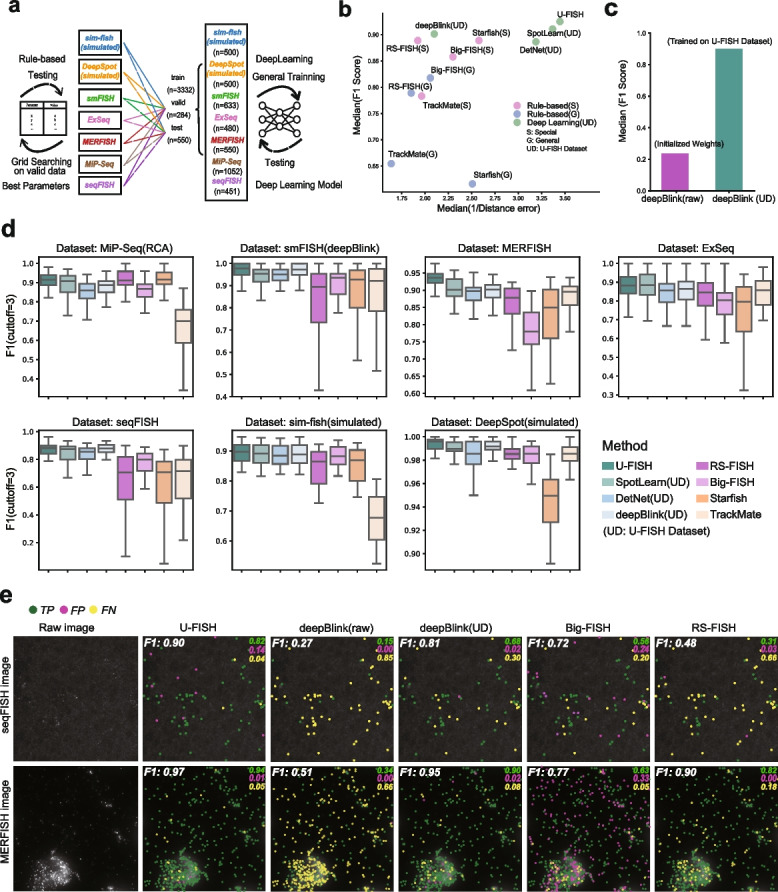
Comparison of spot detection methods. **a** Schematic of the benchmarking strategy. Rule-based methods use grid search on validation data to find optimal parameters, which are then applied to test datasets. Deep learning models are trained and validated on separate datasets before testing. **b** Performance comparison of spot detection methods based on median F1 score and median distance error. Deep Learning (UD) represents deep learning models that have been trained on the U-FISH dataset. Rule based (S) refers to the best parameters found through grid search on a specific dataset, while rule based (G) indicates the best parameters found through grid search on the overall dataset. **c** Median F1 scores for deepBlink models. deepBlink (raw) is the model weights pre-trained on smFISH using deepBlink. The deepBlink (UD) model trained on the U-FISH dataset outperforms the deepBlink (raw) model with initialized weights. **d** Box plots displaying the performance of various models across datasets from different sources, measured by the F1 score (cutoff = 3). A higher F1 score indicates a stronger accuracy in spot detection. **e** Visual comparison sample of spot detection methods on seqFISH and MERFISH images. The raw images are compared with results from different methods: U-FISH, deepBlink (raw), deepBlink (UD), Big-FISH, and RS-FISH. true positives (TP) are marked in green, false positives (FP) in red, and false negatives (FN) in yellow. U-FISH shows the highest accuracy with F1 scores of 0.90 for seqFISH and 0.97 for MERFISH, indicating superior detection performance compared to other methods

To benchmark U-FISH’s performance rigorously, we conducted a detailed comparison using various datasets, including experimental and simulated data. For quantitative performance comparison, we used F1 scores and distance errors on the test dataset and compared these metrics against several other methodologies, including both deep learning-based and rule-based approaches (Fig. 2a and the “Methods” section). Remarkably, U-FISH demonstrated much better performance across these diverse datasets without requiring dataset-specific parameter tuning, showcasing its robustness and versatility. In fact, U-FISH achieved the best performance on the majority of datasets compared to other evaluated methods. For the median metric scores, U-FISH achieved an F1 score of approximately 0.924, surpassing deepBlink (F1: 0.901), DetNet (F1: 0.886), SpotLearn (F1: 0.910), Big-FISH (F1: 0.857), RS-FISH (F1: 0.888), Starfish (F1: 0.889), and TrackMate (F1: 0.783). Regarding distance error, U-FISH recorded a value of 0.290 pixels, outperforming all competitors, indicating its high precision in spot localization (Fig. 2b). Moreover, we noticed the value of the U-FISH dataset for deep learning models. After training with the U-FISH dataset, DeepBlink shows a significant improvement in accuracy compared to using the original weights (Fig. 2c). For more detailed benchmarks on all datasets shown in Fig. 2c and Additional file 1: Fig. S2a. Some specific examples of the benchmark are shown in Fig. 2d and Additional file 1: Fig.S3. Furthermore, our tests on datasets with simulated noise revealed that U-FISH possesses strong noise resistance capabilities (Additional file 1: Fig.S2i–k), highlighting its suitability for analyzing FISH images under various experimental conditions and diverse samples from different biological origins.

Considering that the U-FISH method is essentially a type of image enhancement specialized for signal spot detection, we compared it with traditional image enhancement methods such as deconvolution and neural network-based image restoration methods such as CARE [19]. The results show that U-FISH enhanced images achieve better performance for signal spot detection (Additional file 1: Fig.S4b) and the enhanced images produced have a higher signal-to-noise ratio(Additional file 1: Fig.S4c).

Lastly, U-FISH’s optimized and compact network architecture, comprising only 163k parameters (Additional file 1: Fig.S5a), contributes significantly to its computational efficiency. This streamlined design ensures that U-FISH maintains exceptional accuracy and achieves superior processing speeds, particularly on devices equipped with GPUs, making it an ideal choice for high-throughput analysis. Its commendable efficiency extends to CPU-based systems as well (Additional file 1: Fig.S2e,f), demonstrating the model’s versatility across various computing environments. The compact size of the U-FISH network, with an ONNX file of merely 676kB, underscores the ease with which it can be deployed, even in resource-constrained settings. This level of computational performance and small footprint underpin U-FISH’s capability to handle large images effectively and broadens its applicability across a variety of scenarios, including deployment in web browsers. The combination of high efficiency, low parameter count, and minimal storage requirements makes U-FISH a highly deployable solution, suitable for a wide range of applications from desktop-based analysis to cloud and web-based platforms.

### Application in imaging based spatial-omics data analysis

**Fig. 3:**
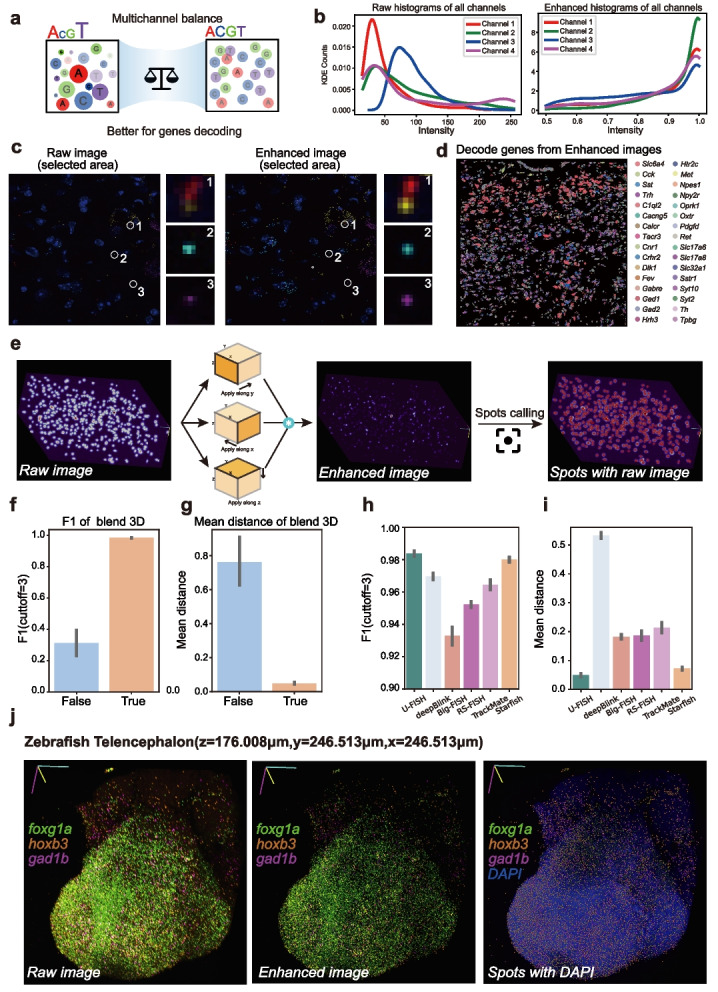
U-FISH applications in imaging-based spatially resolved transcriptomics data analysis. Enhancement and decoding capabilities of U-FISH in spatially resolved transcriptomics with multi-color 3D interpretation. **a** The enhance function of U-FISH effectively balances the intensity and size of signal points in the image, facilitating multi-channel decoding. **b** Histograms of the intensity of identified signal points in the center pixels across four channels in raw and enhanced images during round1. **c** Display of raw and enhanced images. Post-enhancement, images exhibit more uniform and structured signal characteristics, facilitating more precise analysis. **d** Gene coordinates decoded from the enhanced images. **e** schematic representation of the 3D blending strategy used for volumetric images. In this approach, the U-FISH model (trained on 2D data) is applied along the three axes (x, y, z) for image enhancement. The result images from each axis are then multiplied to reconstruct a 3D enhanced image. **f**, **g** The accuracy of the results obtained using the 3D blending strategy against enhancement performed solely along the z-axis. Here, **f** focuses on the F1 score metric, and **g** on the distance error metric. **h**, **i** The performance of different methods on a 3D benchmark using simulated datasets. **h** The differences in F1 scores. **i** The variations in distance errors among the methods. **j** 3D view of the expression and distribution of the foxg1a, hoxb3, and gad1b genes detected by MiP-Seq in the whole mount of zebrafish telencephalon and the enhanced imaging results and the spots identified by U-FISH

U-FISH technology can be effectively applied in spatially resolved transcriptomics data analysis. One significant advantage of U-FISH is its capability for accurate and universal identification of signal points, a common requirement in imaging-based spatial omics. Moreover, U-FISH addresses the growing demands of spatial omics, such as increasing gene throughput through encoding across different channels and even across rounds, which complicates decoding and the challenges of signal point recognition in 3D image data. The image enhancement features of U-FISH offer new possibilities for overcoming these challenges. Initially, this study demonstrates the crucial role of U-FISH in spatial transcriptomics data analysis through a slice along the midline of the dorsal raphe nucleus in the mouse brain using MiP-Seq technology [10]. Through a three-round encoding process, 32 genes were encoded (Additional file 1: Fig.S6b), with each channel representing one gene in the first round. In the second and third rounds, eight channels were paired to encode 28 genes through barcode combinations. To balance these discrepancies caused by fluorescence probes and imaging equipment stability, U-FISH’s enhancement capability effectively addresses these issues, scaling the peak intensity of fluorescent signal points in all channels from various values to 1.0 (Fig. 3a, b and Additional file 1: Fig.S6a).

Building on this, scatter plots of gene expression visualized in situ (Fig. 3d) revealed consistency in the distribution of several genes, including Trh, Gad1, Npas1, Ret, Syt2, C1ql2, Met, and Tacr3, which are also studied in Jing Ren et al.’s (2019) research [27]. Additionally, the distribution of cell types defined by marker gene expression, such as serotonin (5-HT) neurons (Additional file 1: Fig.S6e), aligns with previous studies. These results prove that U-FISH can accurately identify fluorescent signal points in images and that the U-FISH enhancement leads to high accuracy in decoding based on the balanced intensity of multi-channel signal points.

To further elucidate the significant role of U-FISH enhancement in balancing fluorescence intensity across channels during decoding, we also compared the results obtained from extracting features from raw images (Additional file 1: Fig.S6c,d). Since there is no ground truth, it is challenging to evaluate the superiority of either method directly. We compared the number of genes decoded using raw and enhanced images, and found that, with the same input spots, using enhanced images resulted in more genes being decoded (Additional file 1: Fig.S6f). Moreover, by calculating the Moran’s *I* score, we show that for most genes, the spatial distribution of decoded genes from enhanced images is more spatially clustered(Additional file 1: Fig.S6g). These metrics indicate that using U-FISH to enhance different channels can improve the decoding performance. In addition, to further demonstrate the advantages brought by using U-FISH enhanced image decoding, we applied U-FISH enhanced images to decode STARmap data(160 gene set) [28] with the starfish pipeline [14]. The results indicate that based on the U-FISH enhanced image decoding, compared to the original Starfish pipeline(Additional file 1: Fig.S7a), it is able to decode approximately 2.5 times more gene spots(Additional file 1: Fig.S7b) while preserving the original spatial distribution patterns(Additional file 1: Fig.S7c–e).

An advancement presented by U-FISH is the extension of deep learning-based spot detection methods to 3D imaging, which had not been well resolved before due to certain inherent challenges. One obstacle to applying deep learning approaches to 3D FISH images for spot detection is the difficulty in obtaining high-quality annotated 3D datasets. This challenge is primarily attributed to the labor-intensive process of manually identifying center points within the 3D space. The workload required for accurate annotation in 3D is significantly greater than that for 2D images, complicating the acquisition of reliable 3D training data. Additionally, there is a need for efficient implementations that minimize GPU memory requirements for model training, further complicating the transition to 3D. These challenges underscore the necessity of developing a strategy that enables models trained on 2D data to be effectively applied to 3D imaging scenarios, thereby overcoming the hurdles of data annotation and computational demands.

A straightforward approach to applying a 2D model to 3D data involves sliding the model along the *z*-axis to perform inference. However, since these models are not trained specifically on 3D data, they might inadvertently enhance signals not central to the *z*-axis. This issue can lead to the elongation of signals along the *z*-axis, potentially causing repetitive detection of a single point when applying downstream detection algorithms. To address this issue, we implemented a new approach, which involves applying 2D models along the three axes (*x*, *y*, and *z*) to enhance the image, resulting in three separate image stacks. These stacks are then multiplied together to reconstruct a 3D enhanced image (Fig. 3e). Experimental results have demonstrated that this method significantly improves detection accuracy on 3D simulated datasets (Fig. 3f, g). Furthermore, comparative analysis with specific benchmark numbers indicates that U-FISH significantly outperforms other methods in terms of accuracy when applied to simulated datasets. For the mean metric scores, U-FISH achieved an F1 score (cutoff = 3) of 0.984 and a distance error of 0.049, demonstrating superior performance in spot detection accuracy and precision of localization. In comparison, deepBlink recorded an F1 score of 0.970 and a distance error of 0.533, while Big-FISH showed an F1 score of 0.933 and a distance error of 0.182. RS-FISH and TrackMate also trailed behind with F1 scores of 0.952 and 0.964, and distance errors of 0.186 and 0.213, respectively. Notably, Starfish came close with an F1 score of 0.980 and a distance error of 0.072. These results, illustrated in Fig. 3h and i, underscore U-FISH’s advanced capability in detecting FISH signals with remarkable accuracy and minimal localization error, setting a new benchmark for performance in simulated 3D datasets.

To vividly demonstrate the practical effectiveness of U-FISH on 3D data, we utilized images of the Zebrafish Telencephalon from the MiP-Seq study [10]. These images consist of three signal channels—foxg1a, hoxb3, and gad1b—alongside one DAPI staining channel (Additional file 1: Fig.S6h). The dimensions of the images are $z = 176.008\,\mu \text {m}$, $y = 246.513\,\mu \text {m}$, and $x = 246.513\,\mu \text {m}$. Using U-FISH, we enhanced the signal-to-noise ratio of the three signal channels (Additional file 1: Fig.S6i,j), performed signal point detection for each gene, and visually displayed the results in Napari (Fig. 3j). This approach highlights U-FISH’s capacity to enhance and identify gene signals in complex 3D datasets accurately.

Building on the innovative approach for 3D spot detection, U-FISH also employs a strategic method for handling large-scale multidimensional-image inference, particularly crucial for large images stored in formats such as OME-Zarr [29]. This strategy involves reading and processing individual data blocks from the Zarr file, one at a time, and then storing the processed results back onto another Zarr file. The process continues by sliding the window to handle the entire dataset sequentially. This method minimizes memory usage, making U-FISH highly adaptable and robust across various imaging scenarios. This approach further demonstrates U-FISH’s capability to seamlessly integrate into existing bioimaging workflows, providing a scalable solution for the analysis of extensive FISH imaging data, thereby reinforcing the framework’s utility in advancing the field of FISH analysis.

### Model generalization performance and cross-task transfer learning

The ability to generalize across different domains is crucial for the effectiveness of deep learning models. We compared three training modalities-the General model trained on a diverse set of datasets, the Special model trained on a specific dataset, and the Fine-Tuned model which is the General model further trained on a specific dataset, to assess model performance across diverse datasets. Results show small performance gaps among the three models, with General and Fine-Tuned models outperforming the Special model in some datasets (Additional file 1: Fig.S2b). This suggests that training on diverse datasets enhances model versatility. We also conducted a leave-one-out experiment to test generalization on unseen datasets. The results demonstrate that the U-FISH model (mean F1: 0.834, distance error: 0.429) outperforms deepBlink (mean F1: 0.823, distance error: 0.531) in adapting to new, unseen data (Additional file 1: Fig.S2c). This highlights U-FISH’s robustness and potential as a versatile tool for FISH image analysis across various dataset characteristics.

**Fig. 4:**
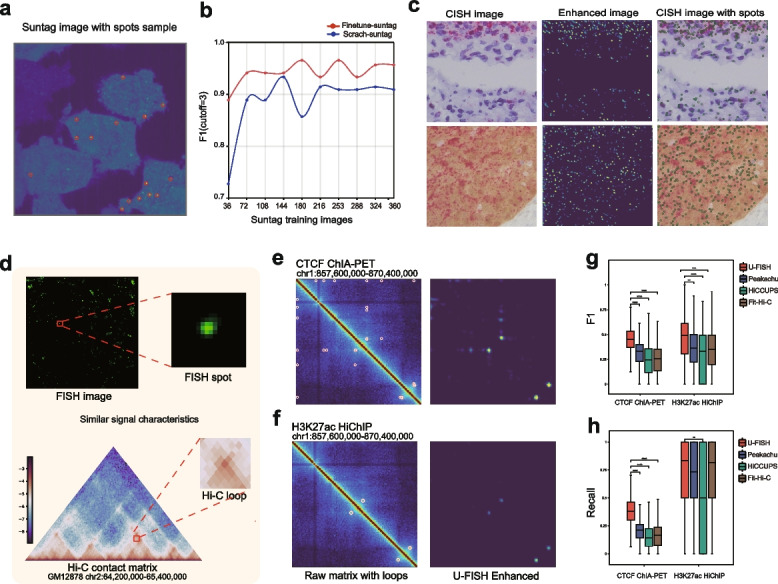
Transfer learning for general spot detection tasks. **a** Suntag image with spots. **b** Graph comparing the performance of models trained from scratch versus those fine-tuned on a general model, as a function of the number of Suntag training images used. The *y*-axis represents the median F1 score, illustrating that fine-tuning requires fewer data to achieve improved performance. **c** CISH raw image, enhanced image and predicted spots. **d** Visualization of the similarity and differences between FISH signal spots and Hi-C contact matrix loops, highlighting local feature comparisons. **e**–**f** Hi-C contact matrices for two chromatin regions, depicting ground truth interactions (CTCF ChIA-PET and H3K27ac HiChIP) and their enhancements post U-FISH model fine-tuning. **g**–**h** Benchmark comparisons of U-FISH with other loop callers, indicating the superior performance metrics of U-FISH in detecting loops within Hi-C contact matrices

To determine the minimum data requirements for training a robust General model, we conducted downsampling tests to explore data requirements for training a General model. Results show satisfactory performance (average F1 score > 0.85) with about 300 training images (512 × 512 pixels). Performance improves further (average F1 score > 0.9) with over 1800 images, showing slow improvement thereafter (Additional file 1: Fig.S2d). To investigate if fine-tuning could reduce required training data, we used SunTag approach images of single RNA translation [30]. These images feature distinct spots but differ from smFISH (Fig. 4c). We found that fine-tuning the General model requires fewer training images than training from scratch to achieve better performance. Notably, only about 30 training images (512 × 512 pixels) were sufficient for good results through fine-tuning, maintaining superior accuracy with increased training images (Fig. 4d). In addition, to demonstrate the broader cross-task learning capability of U-FISH, we fine-tuned the model on a completely different dataset consisting of chromogenic in situ hybridization (CISH) images captured under bright-field microscopy conditions, and found that U-FISH was still able to achieve good predictive performance (Fig. 4e).

The universal effectiveness of the U-FISH model in spot detection tasks suggests its potential for transfer learning to datasets outside of spatial transcriptomics. We have tried to apply U-FISH to a broader range of spot detection tasks other than FISH data. The three-dimensional genome, characterized through High-throughput chromosome conformation capture (Hi-C) [31], features chromatin loops (loops) can be detected using techniques such as ChIA-PET [32] and HiChIP [33]. Upon visualizing the Hi-C contact matrices as images, these loop structures exhibit strong characteristic similarities to the spots observed in U-FISH training data (Fig. 4e, f). This similarity enables the application of U-FISH’s network to develop a deep learning-based model for the identification of Hi-C loops. To construct a dataset for Hi-C loops, we downloaded CTCF ChIA-PET [34] and H3K27ac HiChIP [35] data, serving as ground truth. We fine-tuned the U-FISH model separately on each dataset, using data from chromosome 1 as the test set and the remaining data as the training set. We used the trained model to predict loops in chromosome 1 and visualized the results within the region chr1:857,600,000–870,400,000 (Fig. 4g, h). The results demonstrate that the U-FISH-trained model performs well in the identification of Hi-C loops. For a comprehensive evaluation of U-FISH (Hi-C loops calling), we benchmarked against commonly used software, including Peakachu [36], HiCCUPS [37], and Fit-Hi-C [38]. Since neither HiChIP nor ChIA-PET can comprehensively capture all loops, we assessed the performance using F1 score and recall as metrics. The results indicate that U-FISH significantly outperforms other models in F1 score across both the CTCF ChIA-PET and H3K27ac HiChIP datasets. While the recall did not show a significant difference compared to Fit-Hi-C and Peakachu in the H3K27ac HiChIP dataset, it exhibited better stability, with recall values significantly higher than other methods in general (Fig. 4i, j). This result demonstrates the potential of applying the U-FISH pre-training model with fine-tuning training to a wider and more general spot detection task, excluding FISH data.

### User-friendly tools for facilitating the community adoption

**Fig. 5:**
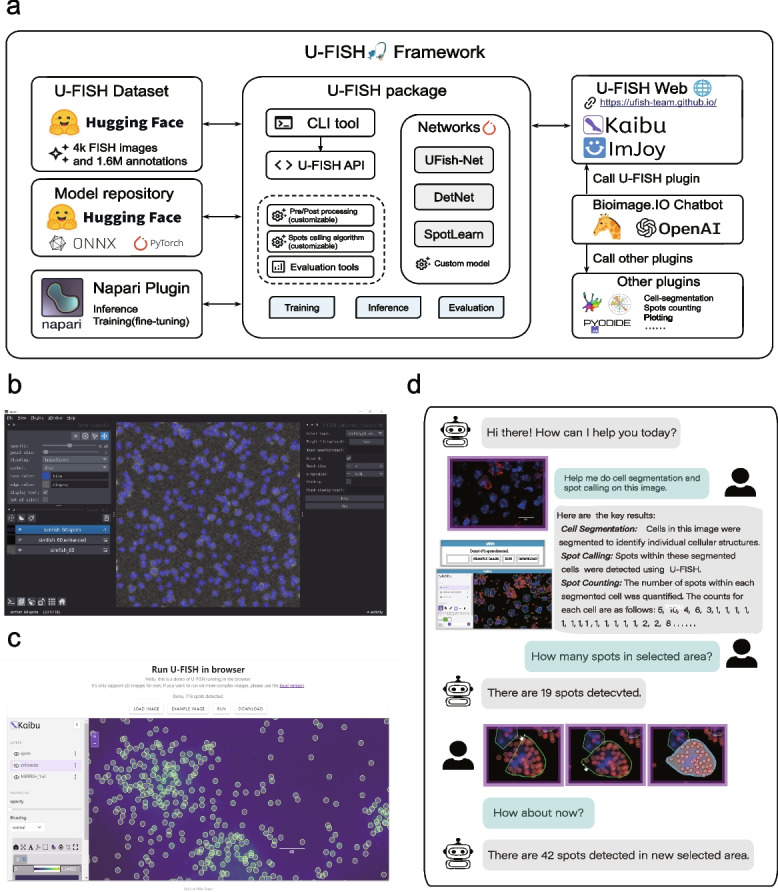
User-friendly tools for facilitating community adoption. **a** The U-FISH Framework provides a comprehensive foundation for the development of future FISH spot identification methods. **b** The napari-ufish plugin features an Inference functionality interface, which, upon activation, displays a panel on the right side of the Napari viewer. This panel allows users to configure various operational options, such as enabling chunking and setting the chunk size. Upon clicking the “Run” button, the plugin employs the U-FISH model to compute and display the enhanced image and corresponding spots within the Napari browser, as illustrated. **c** The web inference interface of U-FISH showcases an implementation using onnxruntime-web to run the U-FISH model directly in the browser. The interface integrates ImJoy [39] and Kaibu viewer for result visualization. It provides a straightforward, interactive user interface for uploading data, initiating the model run, and downloading the results. This web-based solution facilitates easy access and usage of U-FISH, allowing users to leverage its capabilities without the need for extensive setup or specialized software. **d** An example of a user using Chatbot to invoke U-FISH for RNA smFISH signal spot counting analysis

Next, we extended U-FISH from a model to a comprehensive framework for training of FISH spot detection models, evaluating results, and facilitating inference across multiple platforms (Fig. 5a). To enhance accessibility for end-users, we developed a user-friendly graphical user interface (GUI) for U-FISH, aiming to streamline the user experience for both novices and experienced researchers. Our Napari Plugin enabls users to invoke U-FISH for predictions and model training directly through a desktop application (Fig. 5b). In addition to the desktop application, we implemented a U-FISH Web interface, leveraging ImJoy [39], Kaibu, and Webassembly. This innovative approach allows for model predictions to be executed directly within a web browser, significantly lowering the barrier to entry for all users (Fig. 5c). The web interface not only makes easy access to U-FISH’s powerful capabilities but also incorporates a visualization of the training data. More specifically, it permits simultaneous display of FISH images and their UMAP dimensionality reductions providing an interactive interaction with these data (Additional file 1: Fig.S1b).

With the rapid development of large language models (LLMs), their capabilities in natural language understanding and translating natural language into code have significantly enhanced software interactivity and reduced user barriers. Users can perform complex analyses by integrating LLMs with code invocation capabilities simply by conversing with the LLM. To enable users to access U-FISH in a conversational manner for advanced FISH image analysis, we have integrated U-FISH web client, Cellpose, and Web Python console as plugins into the Bioimage.IO Chatbot [40]. By leveraging the power of large language models (LLMs), users can interact with a chatbot to invoke tools such as U-FISH and Cellpose, conduct analyses based on the results, and engage in interactive sessions. We demonstrate a use case where users can perform cell segmentation and spot detection through conversations with the chatbot. Additionally, users can combine GUI operations with chatbot interactions to count spots within selected regions (Fig.5d). This entire process can be conducted within a web interface (Additional file 1: Fig.S8) without installing separate software.

The core components of U-FISH are encapsulated within a Python package, which offers APIs and CLI interfaces for training, prediction, and result assessment. This package includes network architectures for UFish-Net (Our network), DetNet, and SpotLearn, all implemented using PyTorch, and allows for the integration of custom network architectures by users. Importantly, U-FISH supports a wide range of file formats, including TIFF, OME-Zarr [29], and N5, making it versatile for input data types. It is also capable of handling multi-dimensional images, from 2D (x, y) to 5D (tczxy), ensuring comprehensive applicability across various imaging scenarios. To accommodate large-scale imaging datasets, U-FISH is equipped to process large images through block processing, enabling the handling of images that exceed the available memory size. This approach allows for efficient and scalable analysis, even in resource-constrained environments. The U-FISH package facilitates the easy substitution and modification of functions related to image pre-processing and the post-processing of enhanced images via its API. Its modular design not only enhances the reusability of the U-FISH framework but also greatly benefits subsequent research by facilitating methodological improvements and accelerating the deployment process. Hosted on GitHub with extensive documentation and test data, the Python package can be easily installed via pip. Through these interface solutions, U-FISH aims to democratize access to advanced FISH signal detection and analysis, making it more accessible and user-friendly for the scientific community. This inclusivity ensures that U-FISH stands as a formidable tool in the arsenal of researchers seeking to push the boundaries of FISH analysis.

### The application of U-FISH in AI-assisted DNA-FISH disease diagnosis

**Fig. 6:**
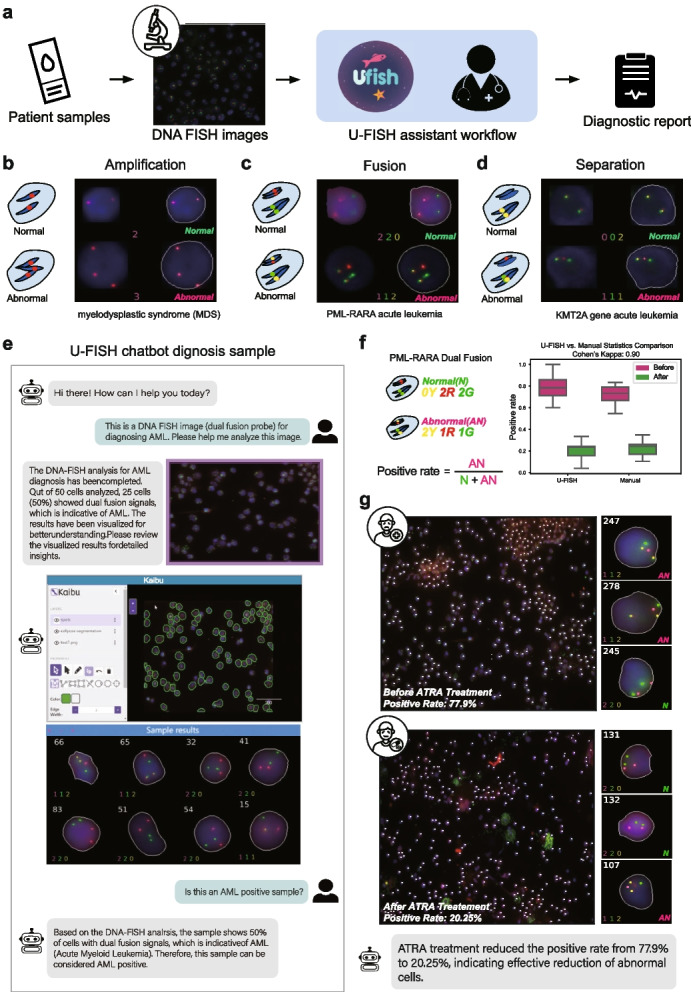
U-FISH Applications in Clinical Diagnostics. **a** Overview of the U-FISH workflow from patient sample collection through DNA FISH imaging to diagnostic reporting, assisting clinicians in detecting genomic abnormalities. **b**–**d** U-FISH diagnostics applied to conditions involving DNA amplification, chromosomal fusion, and separation. It effectively distinguishes normal from abnormal states by counting signal spots, aiding in disease diagnosis associated with genomic structural variations. **e** Calculation of the positive rate for PML-RARA dual fusion by comparing abnormal (AN) cells displaying 2Y, 1R, 1G signals to normal (N) cells with 0Y, 2R, 2G signals, using the formula: $\text {Positive rate} = \frac{\text {AN}}{\text {N} + \text {AN}}$. **f** Box plot illustrating the positive rates measured by U-FISH and expert manual annotation before and after a specified intervention. Both methods display higher positive rates before the intervention, with reduced rates observed after. The comparison demonstrates substantial agreement between the two methods, as indicated by a Cohen’s Kappa coefficient of 0.90 (*N *= 30). **g**, **h**, FISH analysis of cells before (**g**) and after (**h**) ATRA treatment. Prior treatment shows a 77.9% positive rate, significantly reduced to 20.25% post-treatment, indicating a decrease in abnormal cells. Insets magnify selected cells to detail the signal patterns of AN and N cells, with individual cells numbered for reference

DNA FISH signals closely resemble those in imaging-based Spatially Resolved Transcriptomics data. This characteristic similarity enables U-FISH to serve as an excellent base model for identifying DNA-FISH signals (Fig. 6a). On this foundation, a minimal dataset can be constructed to fine-tune the model, thereby quickly and conveniently obtaining a more precise, adapted model.

To demonstrate the application of U-FISH in DNA-FISH data analysis, we collected DNA FISH hybridization images of three hematologic malignancies provided by the clinical laboratory doctors. These images utilized three different types of probes: enumeration probes, dual-fusion probes, and break-apart probes, corresponding to myelodysplastic syndrome (MDS), PML-RARA acute leukemia, and KMT2A gene acute leukemia, respectively. We employed a U-FISH model, fine-tuned for DNA-FISH data, to analyze the results from these three types of probes (Fig. 6b–d). The analysis included counting single-channel or dual-channel fusion signal points according to the detection requirements of each probe and determining the positive cell ratio based on the diagnostic criteria for each probe. The results, verified through visualization, were consistent with clinical doctors’ assessments, with detailed cellular results shown in Additional file 1: Fig.S9a–c.

To make it easier for doctors to use U-FISH as an aid in diagnosis and lower the usage threshold, we have also integrated the U-FISH DNA model and related diagnostic functions as a plugin into the Chatbot, enabling clinical doctors to perform diagnostic analyses seamlessly through conversation (Fig. 6e).

Building on the work above, we analyzed another DNA-FISH dataset from a patient with PML-RARA acute leukemia before and after treatment with all-trans retinoic acid. The analysis revealed that the proportion of positive cells significantly decreased from 77.9% before treatment to 20.2% after treatment (*P*-value=$9.07\times 10^-8$, $N_{ROI}=16$), see Fig. 6f, g. These examples illustrate the potential of U-FISH in assisting clinical doctors with DNA-FISH diagnostics.

## Discussion

We introduced U-FISH, a deep learning-based framework designed to meet the growing demands for sophisticated spot detection in next generation fluorescence in situ hybridization (FISH) technologies in the spatial-omics era. By leveraging a comprehensive dataset, U-FISH showcases improvement in accuracy and generalization ability. This dataset, encompassing experiments and simulations, allows U-FISH to operate with high versatility across various datasets and formats without the need for manual parameter adjustments. Furthermore, its design emphasizes scalability, accommodating large and complex data storage formats and extending its utility to 3D FISH data analysis. And U-FISH’s enhanced images can be well applied to the downstream analysis of imaging-based spatial-omics data, improving the accuracy of signal decoding. To improve community adoption and ensure accessibility, U-FISH is complemented by a suite of user-friendly tools, including the Chatbot plugin, Napari plugin, web application, command-line tool and the API. Specifically, we demonstrated that the chatbot plugin of U-FISH can enable users to complete complex RNA quantification and DNA-FISH diagnostic assistance analysis through conversation alone, greatly reducing the user’s learning curve. Finally, the U-FISH entire dataset is made publicly available, laying a foundation for ongoing and future research in this field.

Despite its significant improvements, U-FISH has several limitations that need further attention. One notable challenge lies in identifying densely packed signal spots, where the current model may struggle to distinguish closely situated points accurately. This limitation underscores the need for further refinement in the algorithm’s ability to resolve high-density areas without sacrificing overall performance(Additional File 1: Fig.S10). Additionally, while we have built a relatively broad dataset, it may still fall short of covering all scenarios encountered in the real world, leading to sub-optimal performance under certain conditions. Moving forward, expanding the dataset and developing tools for real-time annotation and training could lower the barriers to model fine-tuning, addressing these limitations. Moreover, compared to rule-based methods, deep learning approaches, including U-FISH, are susceptible to the “hallucination” problem, where neural networks may produce false positives in certain situations. This issue, coupled with the inherent “black box” nature of neural networks, makes it challenging to address these false positives through parameter adjustments alone. Overcoming this drawback requires a multifaceted approach, including further refining the model’s architecture, enhancing training methodologies, and possibly integrating interpretability features to demystify the decision-making process of the neural network. Addressing these limitations will be crucial for elevating U-FISH’s accuracy and reliability in FISH signal detection and analysis, ensuring its broader applicability and effectiveness in real-world scenarios.

## Conclusions

In summary, U-FISH represents a significant step forward in FISH analysis, offering an adaptable, accurate, and user-friendly solution for researchers. While it pushes the boundaries of what is currently possible in spot detection algorithms, ongoing efforts to address its limitations will be crucial in fully realizing its potential and expanding its applicability to even more challenging datasets. Future enhancements to U-FISH will focus on improving the detection of densely packed spots, extending the framework to incorporate more advanced deep learning models, and enlarging the training dataset to cover a broader range of FISH technologies and experimental conditions. Additionally, efforts will be made to streamline U-FISH’s integration with existing bioinformatics workflows, enabling seamless analysis of FISH data in conjunction with other genomic and transcriptomic datasets. Through these improvements, U-FISH aims to remain at the forefront of innovations in FISH analysis, supporting the scientific community in unraveling the complexities of cellular function and gene expression patterns.

## Methods

### U-FISH dataset construction

This dataset is a comprehensive collection of 4166 fluorescence in situ hybridization (FISH) images, amalgamating real experimental data from a variety of sources including MERFISH, seqFISH, ExSeq, MiP-Seq, and smFISH (Fig. 1). It also encompasses simulated data from sim-fish and DeepSpot [25] simulators.

The initial phase of the process involved training a standard U-Net convolutional neural network (CNN) using the PyTorch framework. Although this model, with its approximately 8 million parameters, was not intended for practical deployment due to its size, it effectively captured the intricate features of FISH signals and served as a pre-labeling tool for the entire dataset. To ensure the highest level of accuracy and reliability, each image, along with its preliminary annotations generated by the U-Net model, was examined and verified by human annotators (Additional file 1: Fig.S1a). This was conducted using the Napari visualization tool [41], facilitating a rigorous review process where automated model outputs were closely aligned with actual ground truths. This comprehensive manual verification and data curation process not only guaranteed the precision of the annotations but also allowed for the organization and association of metadata with each image, providing detailed insights into experimental conditions and source information.

The final U-FISH dataset, a substantial assembly of 4166 images, features a total of 1,638,108 annotations. This rich compilation covers an extensive range of experimental conditions and hybridization targets, making it a valuable resource for developing a generalized FISH point detection algorithm.

### U-FISH model design

Our design draws inspiration from the established image-to-image model architectures, with U-Net [26] being the most extensively utilized among them. This model is renowned for its effectiveness in a diverse array of tasks where image-to-image translation is paramount, providing an excellent foundation for our modified approach.

Following this established protocol, the U-FISH network architecture (UFish-Net) introduces several strategic modifications to the conventional U-Net framework, specifically tailored for fluorescence in situ hybridization (FISH) spot detection (Additional file 1: Fig.S5a). The model has been carefully optimized and compressed, resulting in only 163k parameters, fewer than other comparable models—while maintaining performance (Additional file 1: Fig.S5b). The architecture maintains a configurable channel depth (default: 32 channels) throughout the encoding and decoding stages, which has been empirically determined as an effective trade-off between computational efficiency and model size while preserving feature representation capacity (Additional file 1: Fig.S5c–g).

The encoding pathway consists of convolutional blocks, each containing two consecutive 3$\times$3 convolutions with ReLU activation. For spatial downsampling, UFish-Net employs strided convolutions (3$\times$3 kernels, stride=2) instead of traditional max-pooling operations, enabling learnable feature reduction. Notably, each downsampling operation is followed by a residual block that incorporates 1$\times$1 and 3$\times$3 convolutions with batch normalization, facilitating gradient flow and feature refinement.

At the network’s bottleneck, UFish-Net implements a sophisticated processing module comprising sequential residual blocks with an embedded Convolutional Block Attention Module (CBAM) [42]. This configuration enables the network to adaptively recalibrate both channel-wise and spatial feature responses at the deepest representation level.

The decoding pathway utilizes nearest-neighbor interpolation for upsampling, followed by 3$\times$3 convolutions to refine the upsampled features, effectively mitigating checkerboard artifacts commonly associated with transposed convolutions. Skip connections from the encoding path are concatenated with upsampled features after appropriate padding to handle dimension mismatches. The final decoder block incorporates an additional CBAM module before the output convolutions, ensuring attention-guided feature refinement for precise spot localization.

This architecture achieves an optimal balance between computational efficiency and detection accuracy through its strategic use of residual connections, dual CBAM integration, and learnable downsampling operations, making it particularly well-suited for the demanding task of FISH spot detection in microscopy images.

### Training sample construction and network training

To structure our training database, we allocated the images into distinct sets: 3332 for training, 284 for validation, and 550 for testing. Each data entry is comprised of a pair: an image file in TIFF format and a corresponding spot coordinate file in CSV format. During training, these pairs are read concurrently, with the spot coordinates utilized to produce the target image.

Our experimentation encompassed three distinct methodologies to create these target images: single-pixel representation, morphological dilation, and Gaussian smoothing. The single-pixel target images were crafted by initializing a blank image with identical dimensions to the input image and assigning a value of one to pixels at the spot coordinates. Building upon this, we expanded the marked spots using the skimage.morphology.dilation function, with skimage.morphology.disk(2) as the structuring element. We tested various sizes of the target to optimize the dilation process. For Gaussian smoothing, the single-pixel image underwent filtering with a Gaussian filter set to a sigma value of 1. After smoothing, the resulting image’s intensity was normalized to 0-1. After extensive trials, Gaussian smoothing was chosen as the preferred method for generating target images, demonstrating the most effective outcomes in training efficiency and fidelity to the input data.

The training of UFish-Net was conducted using the Adam optimizer with a learning rate of $1 \times 10^{-4}$ and a batch size of 16. The loss function employed is a hybrid DiceRMSELoss, which combines Dice loss and root mean square error (RMSE) loss in a weighted manner:

$$\begin{aligned} \mathcal {L}_{\text {total}} = \alpha \cdot \mathcal {L}_{\text {Dice}} + \beta \cdot \mathcal {L}_{\text {RMSE}} \end{aligned}$$

where $\alpha = 0.6$ and $\beta = 0.4$ by default. The Dice loss is formulated as:

$$\begin{aligned} \mathcal {L}_{\text {Dice}} = 1 - \frac{2 \sum \nolimits _{i} p_i t_i + \epsilon }{\sum \nolimits _{i} p_i^2 + \sum \nolimits _{i} t_i^2 + \epsilon } \end{aligned}$$

where $p_i$ and $t_i$ represent the predicted and target values respectively, and $\epsilon = 10^{-5}$ serves as a smoothing factor to prevent division by zero. This loss component promotes accurate overlap between predicted and ground truth spot regions.

The RMSE loss component is computed as $\mathcal {L}_{\text {RMSE}} = \sqrt{\text {MSE}(p, t)}$, which ensures pixel-wise accuracy in the predicted density maps. This hybrid loss function effectively balances regional overlap accuracy (via Dice) with pixel-level precision (via RMSE), making it particularly suitable for FISH spot detection where both spatial localization and intensity estimation are crucial.

The training process spans 200 epochs with validation-based model checkpointing, where the best model is saved based on the lowest validation loss. Additionally, periodic model snapshots are saved every 5 epochs. Training progress is monitored through TensorBoard, logging both loss metrics and visual comparisons of input images, ground truth targets, and model predictions. The entire training pipeline was executed on a GeForce RTX 3090 graphics card, with support for multiple optimizer variants including Adam, AdamW, SGD, and RMSprop for experimental flexibility.

### Spot detection on enhanced images

The potency of deep learning models lies in their ability to transform images from varied sources into a representation with consistent features, herein referred to as enhanced images. Given this uniformity, spot detection on enhanced images permits the employment of relatively straightforward and parameter-fixed methods. The choice of spot detection technique is contingent upon the method used for generating target images during the training of the model.

For models trained with single-pixel and Gaussian-smoothed target images, we identify local maxima on the enhanced images using the skimage.morphology.local_maxima function. After this, a fixed threshold (default 0.5) is applied based on the intensity values at the identified coordinates to sieve through the potential spots, filtering out those below the threshold.

Conversely, for models trained on target images processed with dilation, we first convert the enhanced images into binary images using a set threshold(default 0.5). We then detect all connected components within the binary image. Connected components exceeding a certain size threshold—determined by the area of the structuring element used during dilation—are further segmented using the watershed algorithm. The centroid is calculated and designated as the spot’s coordinates for each identified connected component.

This methodology affords a streamlined approach to spot detection, circumventing the need for extensive parameter tuning and enabling consistent performance across datasets derived from disparate imaging conditions.

### The creation of simulated datasets

The data collected comprises both simulated and real experimental datasets, encompassing seven categories in total. Simulated data includes sim-fish and DeepSpot datasets. The sim-fish data is generated using the Sim-FISH python package, which is part of the FISH-Quant v2 [15] framework. The function simfish.simulate_images() is utilized for simulation, with different values set for each parameter to vary the signal noise and the number of signals in the images. The DeepSpot dataset is created using a simulation script provided by the DeepSpot [25], with adjustments made to the parameters. All simulated data consist of images of size 512x512. Detailed parameter information can be found at this link: https://github.com/UFISH-Team/U-FISH/tree/main/data/simulation.

### The source of real experimental datasets

The real experimental data encompasses five categories: seqFISH, ExSeq, MERFISH, smFISH, and MiP-Seq. The seqFISH, ExSeq and MERFISH datasets are sourced from publicly available data at the following URLs, respectively: https://download.brainimagelibrary.org/biccn/zhangl/seqfish/Spinal_Cord_seqFISH_%2301-0001/, https://github.com/dgoodwin208/ExSeqProcessing/wiki/Example-Data-Set, and https://download.brainimagelibrary.org/02/26/02265ddb0dae51de/. The smFISH data is obtained from the deepBlink’s smfish.npz, available at https://figshare.com/articles/dataset/Datasets/12958037. The MiP-Seq data originates from the MiP-Seq [10]. All original images were cropped to a size of 512x512, after which images of low quality, excessive noise or overexposed signals were removed to yield the final dataset required for this study.

### The measurement used in the benchmark

To assess the quality of our results, a critical initial step is the matching of predicted signal points with the ground truth. To expedite this process, we construct a KD-Tree using the coordinates of the predicted points and then query this tree with the ground truth coordinates. This approach efficiently identifies the nearest predicted point for each ground truth signal. If the Euclidean distance between a matched pair (ground truth and predicted point) is less than a predefined cutoff (default value set at 3 pixels), the predicted point is classified as a true positive (TP).

Once the number of TPs is determined, we calculate the false positives (FP) by subtracting the number of TP from the total number of predicted points. Similarly, false negatives (FN) are computed by subtracting the number of TPs from the total number of ground truth points. With these values: TP, FP, and FN, we can calculate the F1 Score: $F1 = 2 \cdot \frac{\text {Precision} \cdot \text {Recall}}{\text {Precision} + \text {Recall}}$ where $\text {Precision} = \frac{TP}{TP + FP}$ and $\text {Recall} = \frac{TP}{TP + FN}$.

In addition to the F1 score, we also calculate the distance error to evaluate the precision of localization. This metric is derived by averaging the distances between all matched pairs of points (ground truth and predicted). The distance error measures the localization error, offering an insight into the model’s performance in accurately pinpointing signal locations. This dual metric calculation approach—focusing on detection accuracy and localization precision—provides a comprehensive assessment of the model’s performance.

### Benchmark for deep learning methods

To evaluate the performance of U-FISH, we conducted a comparative analysis against three deep learning-based models: deepBlink [24], DetNet [22], and SpotLearn [23]. Given the inherent transfer learning capabilities of deep learning models across diverse datasets, we employed a single model trained universally on all datasets to maximize its generalization potential. Notably, we re-implemented these models using the PyTorch framework due to the lack of available implementations that could be readily retrained on our datasets for DetNet and SpotLearn. This re-implementation facilitated the seamless integration of DetNet and SpotLearn into U-FISH’s training and inference framework for comprehensive testing. To ensure a fair comparison, we performed a grid search for DetNet’s hyperparameter $\alpha$ (the sigmoid shift parameter), to identify the optimal setting for the validation dataset. This systematic approach enabled us to tailor DetNet’s performance closely to the specific requirements of our evaluation, ensuring that all models were assessed under equivalent conditions. As SpotLearn only predicts binary masks without actual point localization, the most efficient method for converting the characteristics of SpotLearn’s predicted results into point coordinates is by extracting the centroids of connected components. For DetNet, the predicted results consist of single pixels, and thus, the conversion solely involves mapping these single pixels to coordinates. We applied the skimage.filters.threshold_otsu function to binarize the enhanced output results generated by DetNet and SpotLearn networks. Subsequently, we employed the skimage.measure.label and skimage.measure.regionprops functions to identify signal points within the outputs. To ensure equitable comparisons, particularly considering the tendency for dense signal overlap within SpotLearn’s target process, we integrated a watershed segmentation algorithm. More detailed parameters and training scripts can be found here: https://github.com/UFISH-Team/U-FISH/tree/main/benchmark#deep-learning-methods.

### Benchmark for rule-based methods

U-FISH’s performance was benchmarked against ruled-based methods for single-molecule spot detection in images, including Big-FISH, RS-FISH [16], Starfish [14], and TrackMate [13]. To ensure a fair evaluation, we accounted for the limitation of rule-based methods, which cannot use a single set of parameters to achieve optimal performance across all datasets. Acknowledging this, we adopted a grid search approach to pinpoint the optimal parameters for testing. The evaluation metrics used for comparison were the F1-score and distance error. Detailed grid search parameters and scripts can be found here: https://github.com/UFISH-Team/U-FISH/tree/main/benchmark#rule-based-methods.

### Execution time measurement

Each of the methods was applied to a test dataset consisting of 550 images, each sized at 512 × 512 pixels. Execution time included loading dependencies, loading weights, running the inference process, and logging information. This allowed us to estimate the average time needed for processing a single image. All time measurements were conducted on a workstation equipped with a GPU (NVIDIA GeForce RTX 3090) or a CPU (Intel Xeon 6330). To ensure reliability, each task was repeated five times on both the GPU and CPU to mitigate potential anomalies.

### DR area 32 genes decode

For the 32 genes co-encoded across rounds 1, 2, and 3, we employed the BigWarp plugin in ImageJ (Fiji) [43] for semi-automatic alignment. Additionally, segmentation of the DAPI channel was performed using Cellpose 1.0 [17]. During the decoding of rounds 1, 2, and 3, signal point detection was carried out using the default model of U-FISH, with an intensity threshold set at 0.5. For the 28 genes encoded in rounds 2 and 3, we extracted features from the signal points identified in both raw and U-FISH enhanced images, with all other parameters being constant, and subsequently compared the results. Regarding the decoding process and the computation methods, we have already uploaded the code and associated image data to GitHub and made it publicly available.

### Application of U-FISH in DNA FISH diagnosis

Fluorescence in situ hybridization (FISH) is a pivotal molecular pathology technique that serves as a decision-making tool for physicians in precise tumor classification, prognosis assessment, and treatment guidance. Pathologists are required to manually count probe signals within dozens of cells under a microscope and make diagnostic judgments based on these counts. These complex procedural tasks present significant challenges for pathologists. Consequently, numerous FISH analysis workflows and software have been developed based on artificial intelligence algorithms. These enable accurate identification of cell nuclear contours and automated fluorescence probe counting, even in complex tissues. They are applicable to various types of FISH detection projects, such as breast cancer, gastric cancer, urothelial carcinoma, and colorectal cancer. Additionally, they facilitate the analysis of probe detection results, including gene amplification, fusion, deletion, and breakage.

This study applies U-FISH to DNA-FISH diagnostics in three diseases caused by genomic structural variation, demonstrating its utility in identifying normal and abnormal states.

The analysis consists of three steps: firstly, cell segmentation involves segmenting cells using Cellpose [17] and filtering out low-quality cells. Small cells are removed using the skimage.morphology.remove_small_objects() function. Additionally, closely adjacent cells are filtered based on the distance between their centers, and irregular cells are filtered based on their aspect ratio. Secondly, ROI extraction involves extracting regions of interest (ROIs) based on the masks obtained from cell segmentation. Thirdly, Calling signals and assigning spots for each cell involves using U-FISH to identify fluorescence signals within each ROI. This step generates a signal mask where pixels representing probe signals are set to 1 and applies the skimage.morphology.dilation function to expand the regions containing probe signals. Finally, the signal mask is applied to the original cell image using a given percentile threshold to isolate high-intensity signals. After conversion to masks, adjacent masks are merged into a single mask, and the coordinates are output. Signals from different channels are merged and allocated to different cells using the sklearn.neighbors.KDTree functions to merge signals from different channels and assign them to cells.

### Chromatin loop recognition in Hi-C data

In our study, we employed U-FISH for the recognition of chromatin loops within the three-dimensional genome. The primary datasets utilized for this analysis consisted of 10k resolution Hi-C matrices sourced from the GM12878 cell line, alongside experimentally derived chromatin loops obtained through HiChIP and ChIA-PET methodologies. To facilitate our analysis, we utilized Cooler to transform the 10k resolution Hi-C matrices into multiple training images, each measuring $128 \times 128$ pixels in size. Concurrently, we constructed ground truth labels for these training images by mapping the positions of chromatin loops as delineated in the corresponding loop files onto the training images. Subsequently, we partitioned chromosomes 2 to 22 as our training dataset, reserving chromosome 1 for performance evaluation. This partitioning strategy ensured robust assessment of our model’s generalization capability.

We conducted a comparative analysis of Peakachu, HiCCUPS, and Fit-Hi-C algorithms for loop recognition in chromosome 1 of the GM12878 Hi-C dataset. The evaluation was based on two key performance metrics: F1 score and recall. Given the inherent difficulty in identifying chromatin loops near the diagonal in three-dimensional genome loop detection, as reported in the Peakachu literature [36], we focused our comparison specifically on chromatin loops with a genome span greater than 0.1 megabases (MB). The results for Peakachu, HiCCUPS, and Fit-Hi-C were sourced from the Peakachu publication.

## Supplementary information

Additional file 1: Supplementary Figures.

Additional file 2: Supplementary Tables.

Additional file 3: U-FISH Dataset Metadata Table.

## Data Availability

The U-FISH dataset is publicly accessible on HuggingFace at https://huggingface.co/datasets/GangCaoLab/FISH_spots [44]. This release aims to support and encourage further advancements in FISH analysis research. Some of the real sample data are sourced from public databases, such as smFISH [45], ExSeq [46], MERFISH [47], and seqFISH [48]. The Hi-C contact map of GM12878 is from the NCBI GEO database, accession number GSE63525. The CTCF ChIA-PET interactions in GM12878 were obtained from Tang et al. [34]. The H3K27ac HiChIP interactions in GM12878 were obtained from Mumbach et al. [35]. Results for benchmarking Peakachu, HiCCUPS, and Fit-Hi-C were obtained from J. Salameh et al. [36]. All code related to U-FISH has been made publicly available on GitHub [49] and Zenodo [50]. The U-FISH Python package can be found at https://github.com/UFISH-Team/U-FISH, offering the core functionalities and algorithms. And the code for generating simulated data is also included in this repository. For a graphical user interface experience, the U-FISH Web application is accessible at https://github.com/UFISH-Team/UFISH-Team.github.io, and the U-FISH Napari Plugin is available at https://github.com/UFISH-Team/napari-ufish, facilitating integration with the Napari viewer. Additionally, an online instance of U-FISH Web is hosted at https://ufish-team.github.io/, where users can directly engage in model predictions and dataset exploration without the need for local installation. U-FISH cancer pathology diagnosis code is available at https://github.com/UFISH-Team/U-FISH-cancer-diagnosis. DR area 32 genes decode code is available at https://github.com/UFISH-Team/pDR-area-32gene-decode-example. The code for chromatin loop recognition in Hi-C data using U-FISH is available at https://github.com/UFISH-Team/U-HiC.
