# Supplementary material for: U-FISH: a fluorescent spot detector for imaging-based spatial-omics analysis and AI-assisted FISH diagnosis
Source: Genome Biol. 2025 Sep 1;26:261. doi: 10.1186/s13059-025-03736-x (PMC12400573; doi:10.1186/s13059-025-03736-x)
Supplement: Supplementary file 1 — Additional file 1: Supplementary Figures. [file 13059_2025_3736_MOESM1_ESM.pdf]

Supplementary Figures

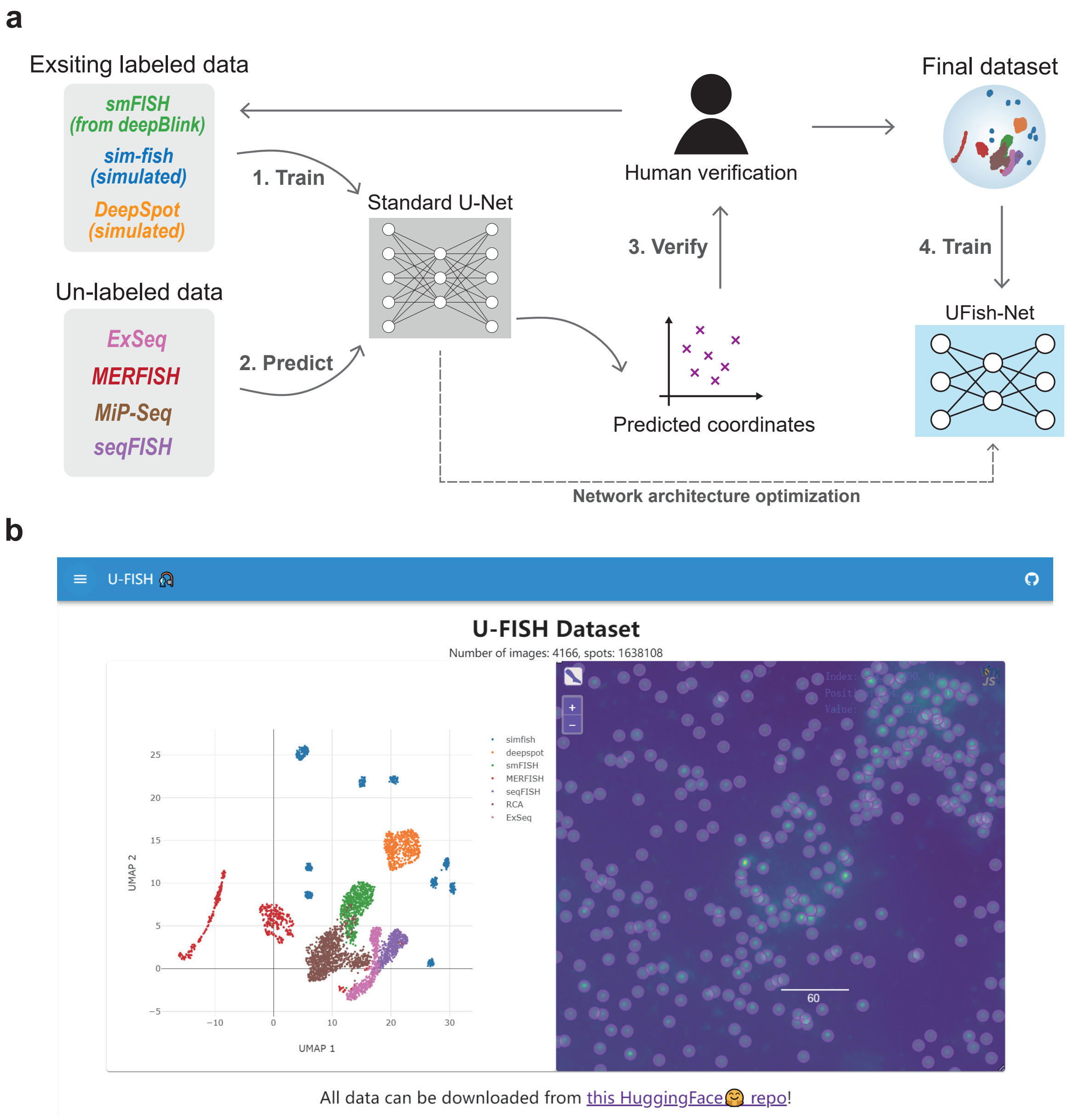

Fig. S1: **a,** Workflow for constructing the U-FISH dataset: A standard U-Net is initially trained on labeled data, including simulations and smFISH datasets. It then predicts coordinates on unlabeled datasets, which are verified and corrected by human annotators. This refined dataset trains the optimized UFish-Net architecture, enhancing predictive performance. **b,** The U-FISH Web interface facilitates dataset browsing and exploration. An interactive scatter plot (left) shows dimensionality-reduced images of the U-FISH dataset, and the corresponding image details are viewable in the Kaibu viewer (right) when a plot dot is selected. This provides a dynamic and intuitive way for users to engage with the data.

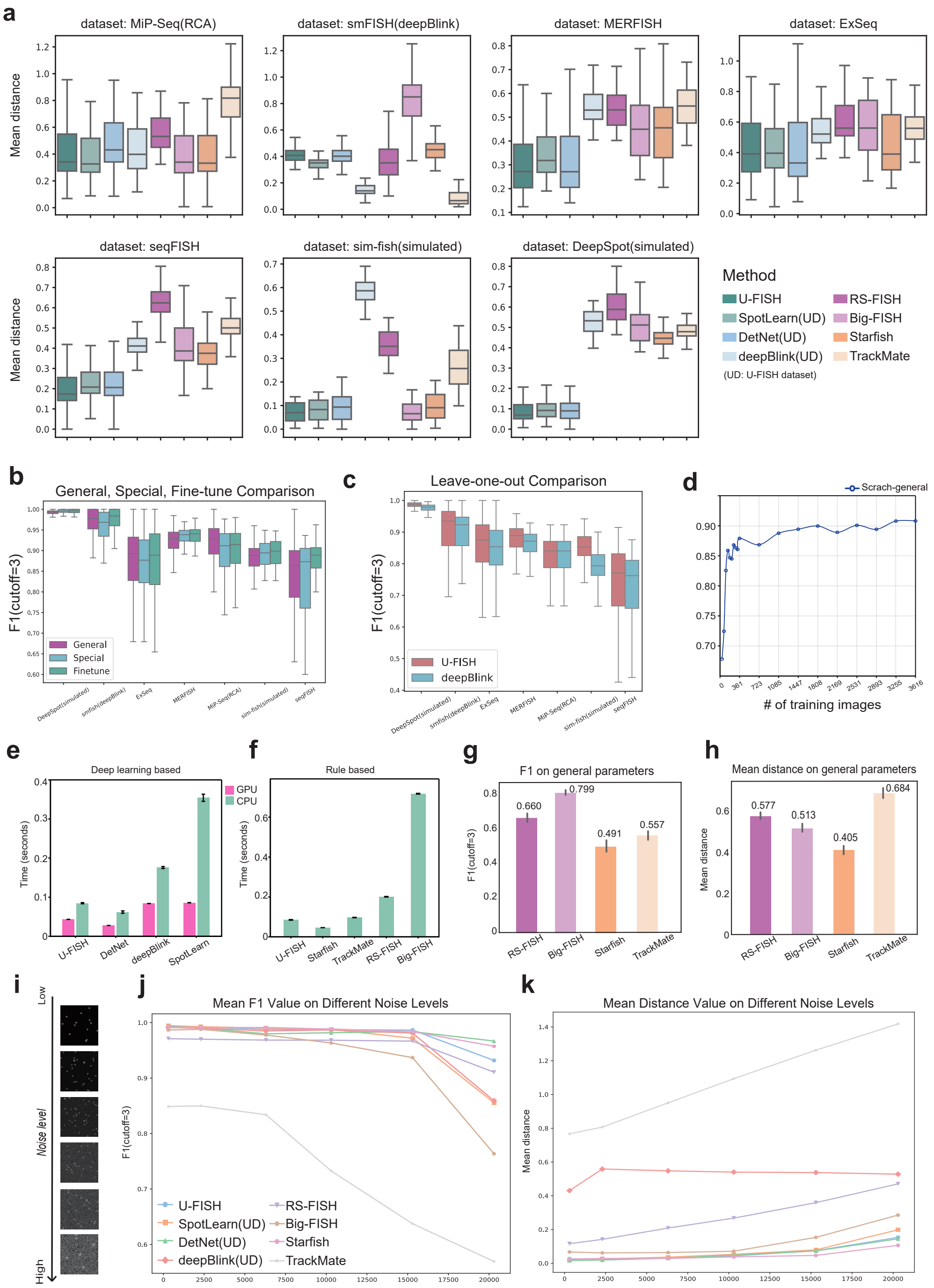

Fig. S2: **a,** Box plots show distance error metrics for various models across different datasets, highlighting U-FISH's precision in spot detection. **b,** Performance evaluation of General, Special, Fine-tune models. **c,** Performance evaluation of U-FISH and deepBlink (trained on U-FISH Dataset) methods using a leave-one-out approach on various datasets, demonstrating their generalization capabilities. **d,** Relationship graph between training data volume and model performance, demonstrating significant gains in F1 score with increased data. **e, f,** Comparison of processing times for deep learning and rule-based methods, differentiating CPU and GPU performances. **g, h,** Rule-based methods' performance on uniform parameters across datasets, emphasizing challenges in parameter adaptation. **i-k,** Robustness of spot detection methods against varying noise levels, illustrated through noise gradients and performance metrics.

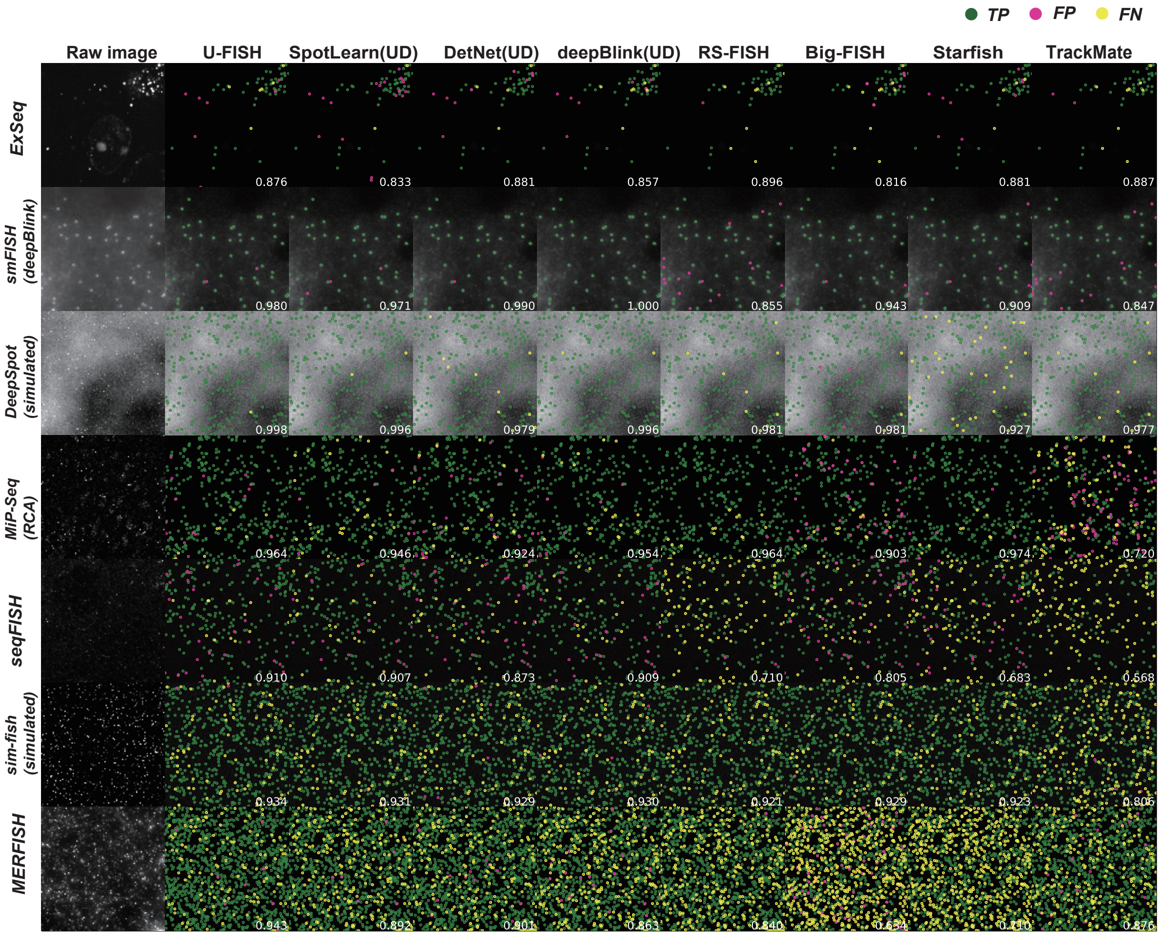

Fig. S3: Benchmark examples with evaluation. This figure presents a series of example results from the benchmark tests, each representing different model and data type combinations. Within each example, the spots are annotated to distinguish between True Positives (TP), False Positives (FP), False Negatives (FN).

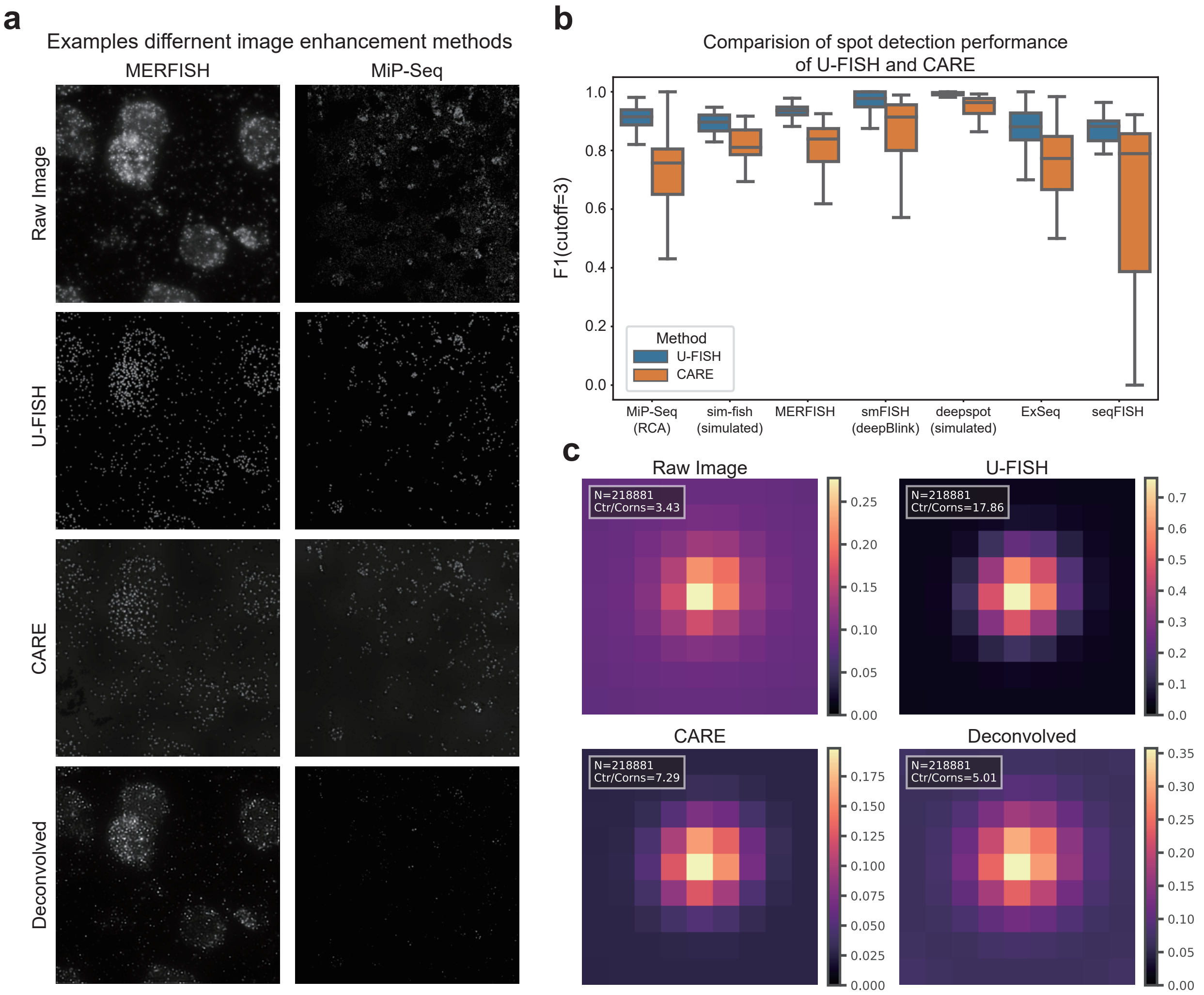

Fig. S4: Comparison of the effects of different image enhancement methods on FISH signal spot recognition. **a,** Examples of two original images, U-FISH enhanced images, CARE enhanced images, and deconvolution images. It is clear that the U-FISH enhanced images have a higher signal-to-noise ratio. **b,** Comparison of the performance of signal spot recognition using U-FISH enhanced images and CARE enhanced images with the same parameters. **c,** The pileup results of the signal spot regions in the raw image and the three types of enhanced images show that the signal in the U-FISH image is clearer, and the ratio of the central point to the four corner positions (Ctrl/Corns) is higher. This indicates that the U-FISH enhanced image has a higher signal-to-noise ratio.

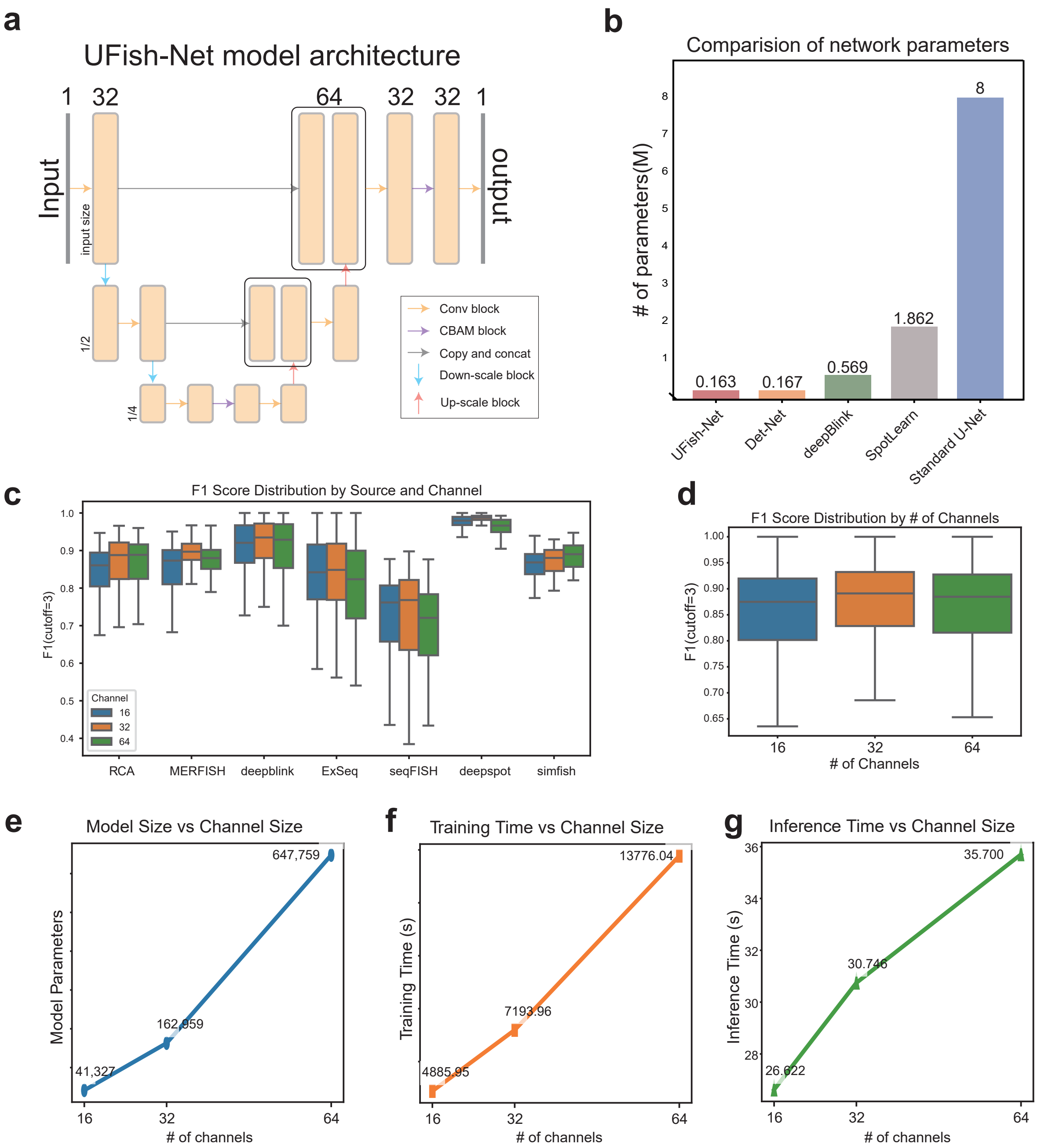

Fig. S5: Comparison of Model Architectures and Performance with Different Numbers of Convolution Channels. **a,** UFish-Net model architecture. **b,** Parameter comparison among different neural network methods. **c,d,** Performance comparison of spot detection with different parameter counts. **e,** Model size under different channel numbers. **f,** Training speed of models with different channel numbers. **g,** Inference speed of models with different channel numbers.

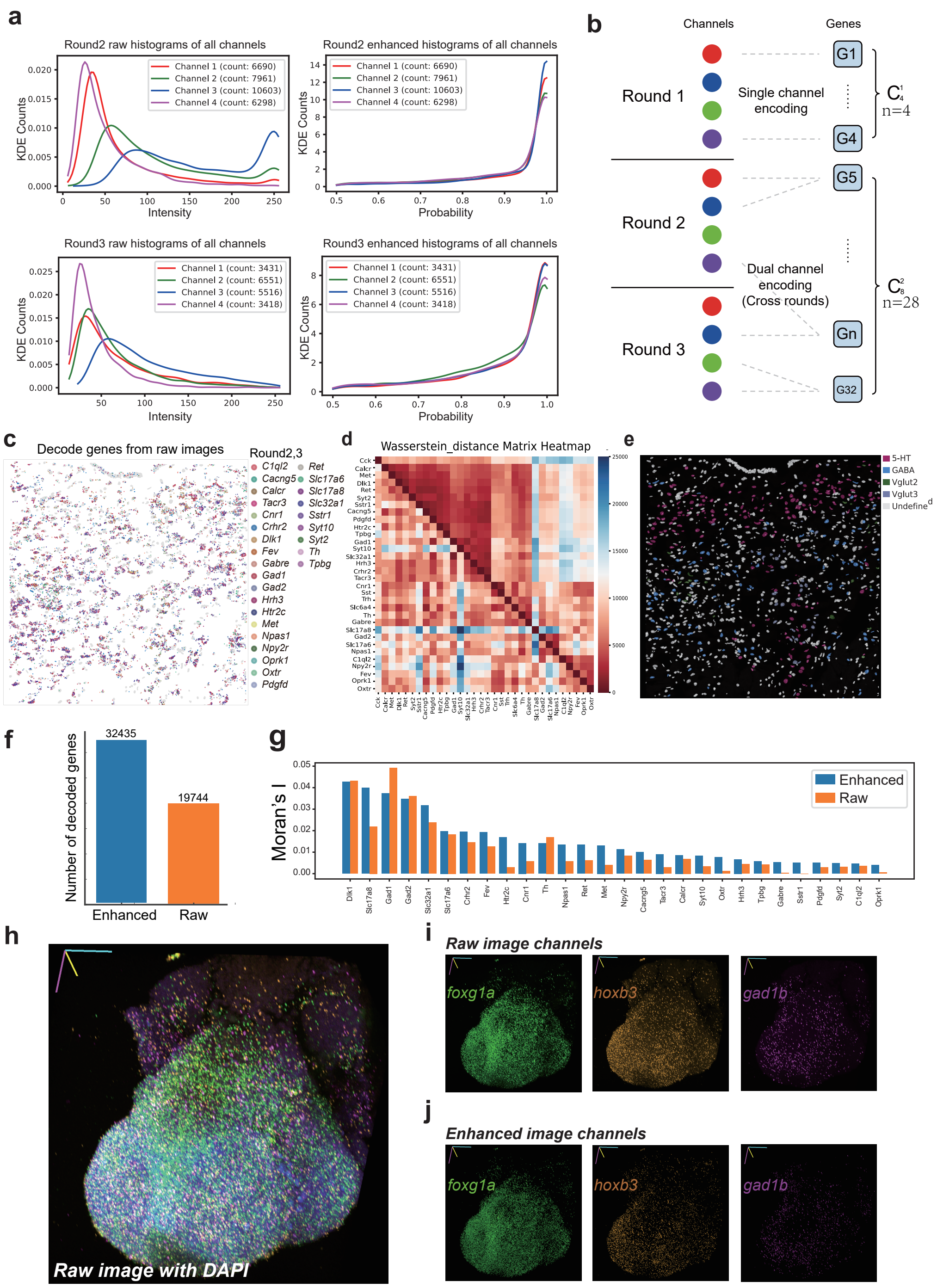

Fig. S6: Enhancement and Decoding Capabilities of U-FISH in Spatially Resolved Transcriptomics with Multi-color 3D Interpretation. **a,** Intensity histograms of signal points across four channels in raw and enhanced images during U-FISH rounds 2 and 3. **b,** MiP-Seq encoding scheme over three imaging rounds: initial round encodes four genes; subsequent rounds use a dual channel approach to encode twenty-eight genes. **c,** Scatter plots of 28 gene expressions from rounds 2 and 3, overlaid on DAPI-stained segmentation results. **d,** Wasserstein distance heatmap comparing gene expression distributions between raw and enhanced image features. **e,** Neuronal cell type annotation based on expression of marker genes 5-HT, GABA, VGLUT2, and VGLUT3, with unclassified neurons labeled as undefined. **f,** Bar graph of decoded genes for U-FISH enhanced image and raw image. **g,** Bar plot showing Moran's I index of different genes's spatial distribution decoded from U-FISH enhanced image and raw image. **h,** Original Zebrafish Telencephalon image with three signal channels and DAPI staining. **i, j,** Comparison of signal channels in Zebrafish Telencephalon before and after U-FISH enhancement.

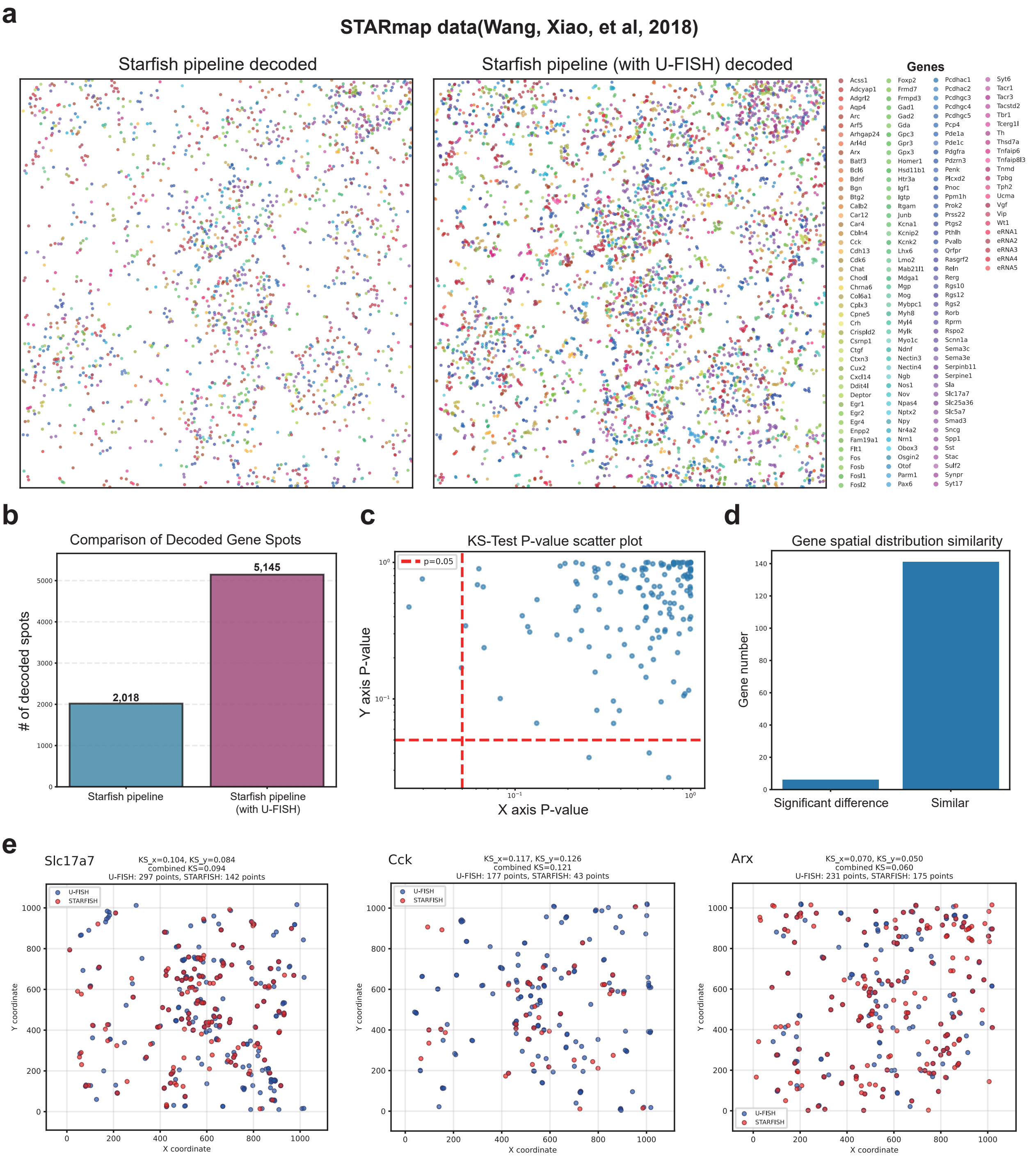

Fig. S7: Comparison of gene decoding performance between Starfish pipeline and STARFISH pipeline with U-FISH integration on STARmap data. **a,** Spatial distribution of decoded gene spots in STARmap data (Wang, Xiao, et al, 2018). The left panel shows results from standard Starfish pipeline decoding, right panel shows results from Starfish pipeline integrated with U-FISH. Each color represents a different gene (160 genes total), with the color legend shown on the right. **b,** Quantitative comparison of decoded gene spots between the two methods. STARFISH pipeline alone decoded 2,018 spots, while Starfish pipeline with U-FISH integration decoded 5,145 spots, representing a 2.5-fold increase in detection sensitivity. **c,** Kolmogorov-Smirnov test p-value scatter plot comparing spatial distributions of genes between the two methods. Each point represents one gene, with X-axis and Y-axis p-values indicating statistical significance of spatial distribution differences. Red dashed lines mark the significance threshold ($p = 0.05$). Most genes cluster above the significance threshold, indicating similar spatial patterns between methods. **d,** Summary statistics of gene spatial distribution similarity. The majority of genes (approximately 140 out of 160) show similar spatial distributions between the two methods, while only a small fraction show significant spatial differences. **e,** Representative examples of spatial distribution patterns for three genes: Slc17a7 (left), Cck (middle), and Arx (right). Blue points represent U-FISH results, red points represent Starfish results. The overlapping distributions demonstrate consistent spatial localization patterns between methods, with KS statistics and combined scores indicating high spatial concordance.

a

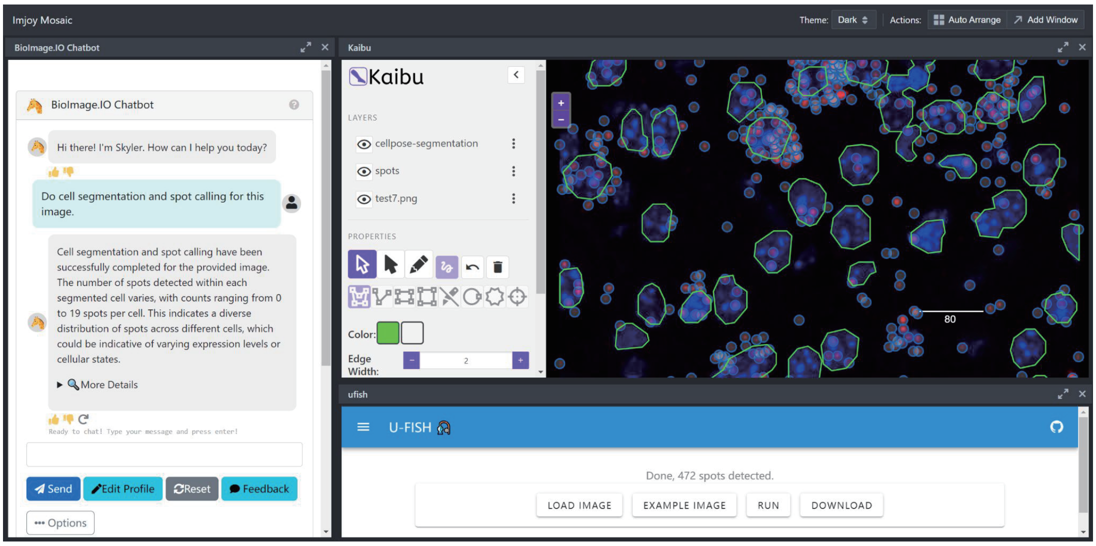

Fig. S8: Graphical User Interfaces. An example of using the Bioimage.IO chatbot and U-FISH web together, users can complete spot detection, cell segmentation, and intracellular spot counting functions through dialogue alone.

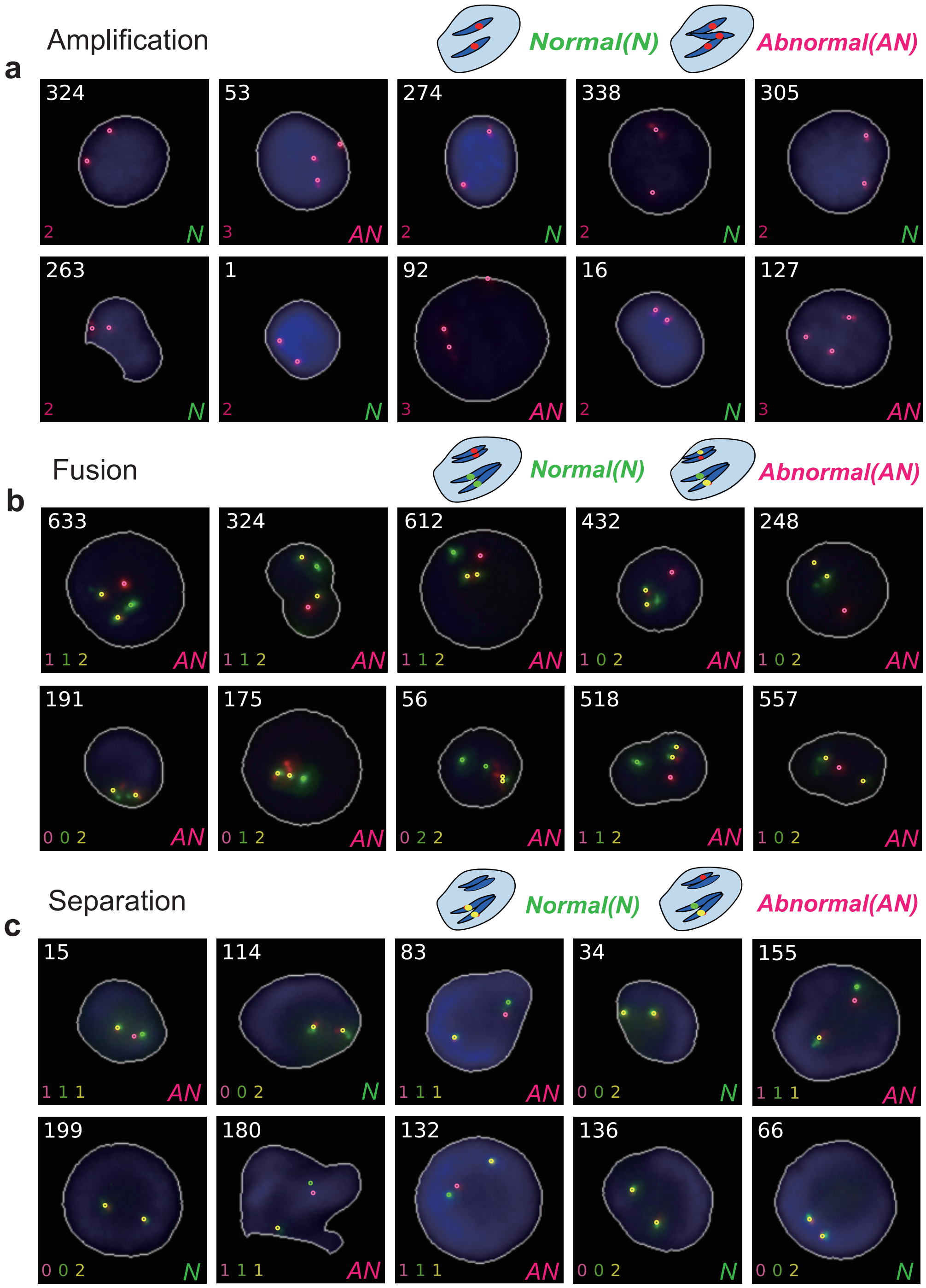

Fig. S9: U-FISH DNA-FISH diagnostics illustrating genomic structural variations across different disorders. **a,** Analysis of myelodysplastic syndrome (MDS) cells, distinguishing normal (N) from abnormal (AN) states based on signal amplification: Normal cells show 2 red signals (2R), while abnormal cells display 3 red signals (3R). **b,** FISH analysis for PML-RARA acute leukemia showing fusion signals: Normal cells exhibit 0 yellow (0Y), 2 red (2R), and 2 green (2G) signals, whereas abnormal cells show 2 yellow (2Y), 1 red (1R), and 1 green (1G) signals. **c,** Examination of KMT2A gene in acute leukemia with separation signals: Abnormal cells are identified by 1 yellow (1Y), 1 red (1R), and 1 green (1G) signals, contrasting with normal cells which show 0Y, 2R, and 2G signals. These panels effectively demonstrate the utility of U-FISH in identifying and distinguishing normal and abnormal cellular states in clinical diagnostics.

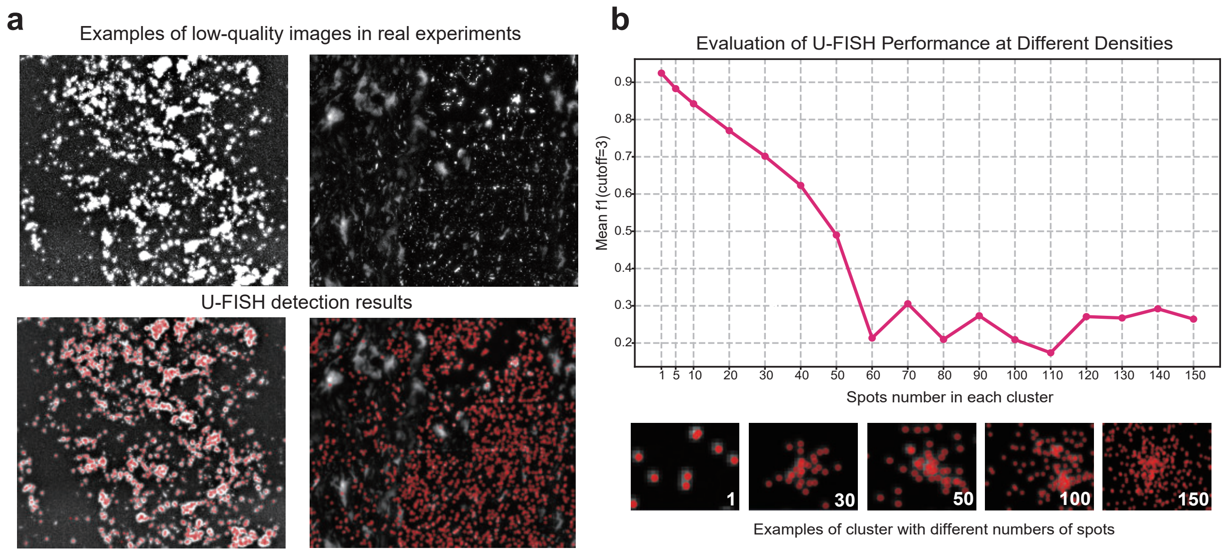

Fig. S10: **a,** Shows low-quality images encountered in real experiments and the detection results of U-FISH on them. **b,** As the signal density of spot clusters increases, the U-FISH recognition accuracy decreases. In the upper panel, the X-axis represents the parameter "spot number in each cluster" used in simfish simulation, and the Y-axis represents the F1 score of U-FISH recognition. The lower panel shows example images with different cluster densities.

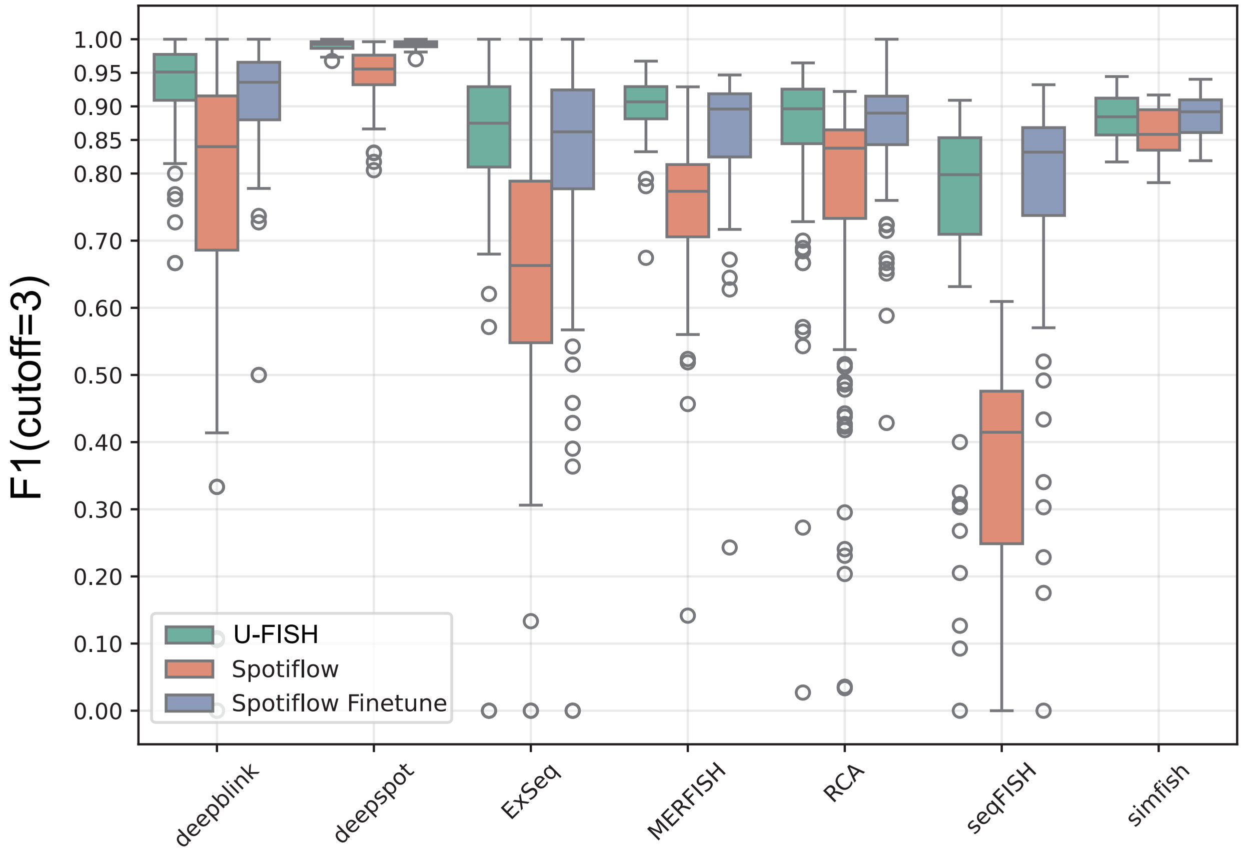

Fig. S11: Performance comparison with Spotiflow on the U-FISH dataset

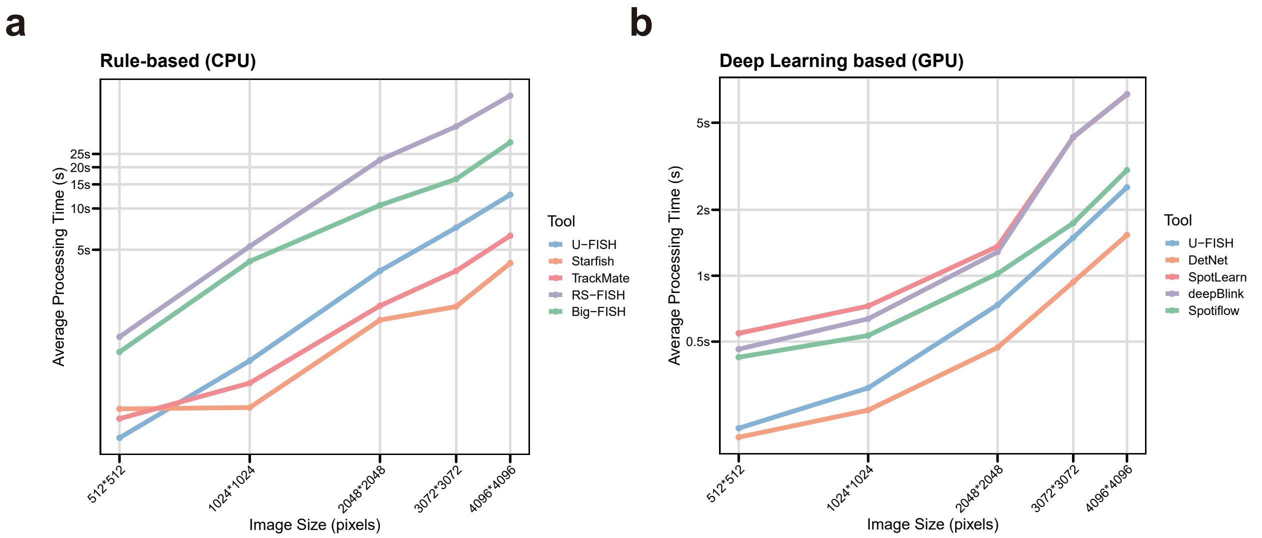

Fig. S12: Comparison of inference computing performance: running speed of ReLU-based methods and deep learning-based methods on CPU and GPU devices for different image sizes.

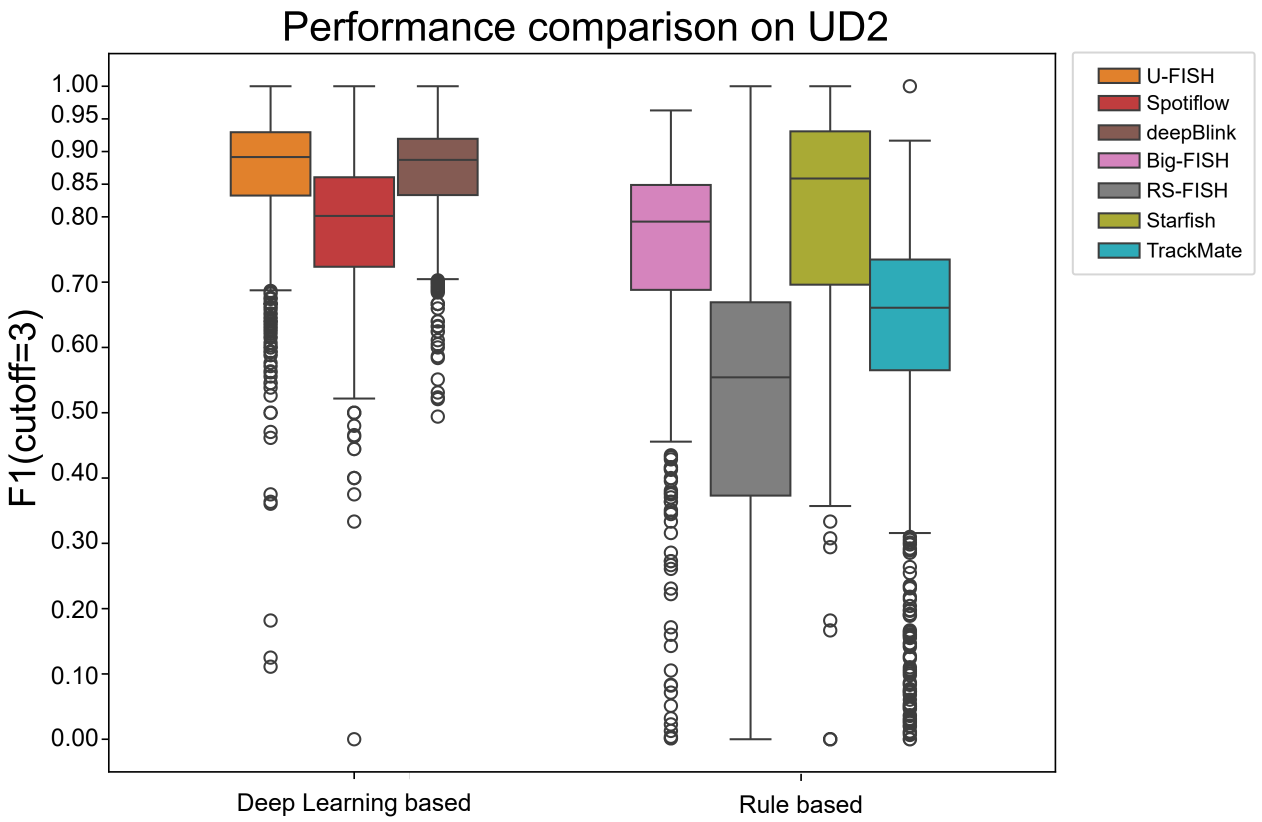

Fig. S13: Evaluation results on an additional dataset comprising multiple species, organs, and tissue types

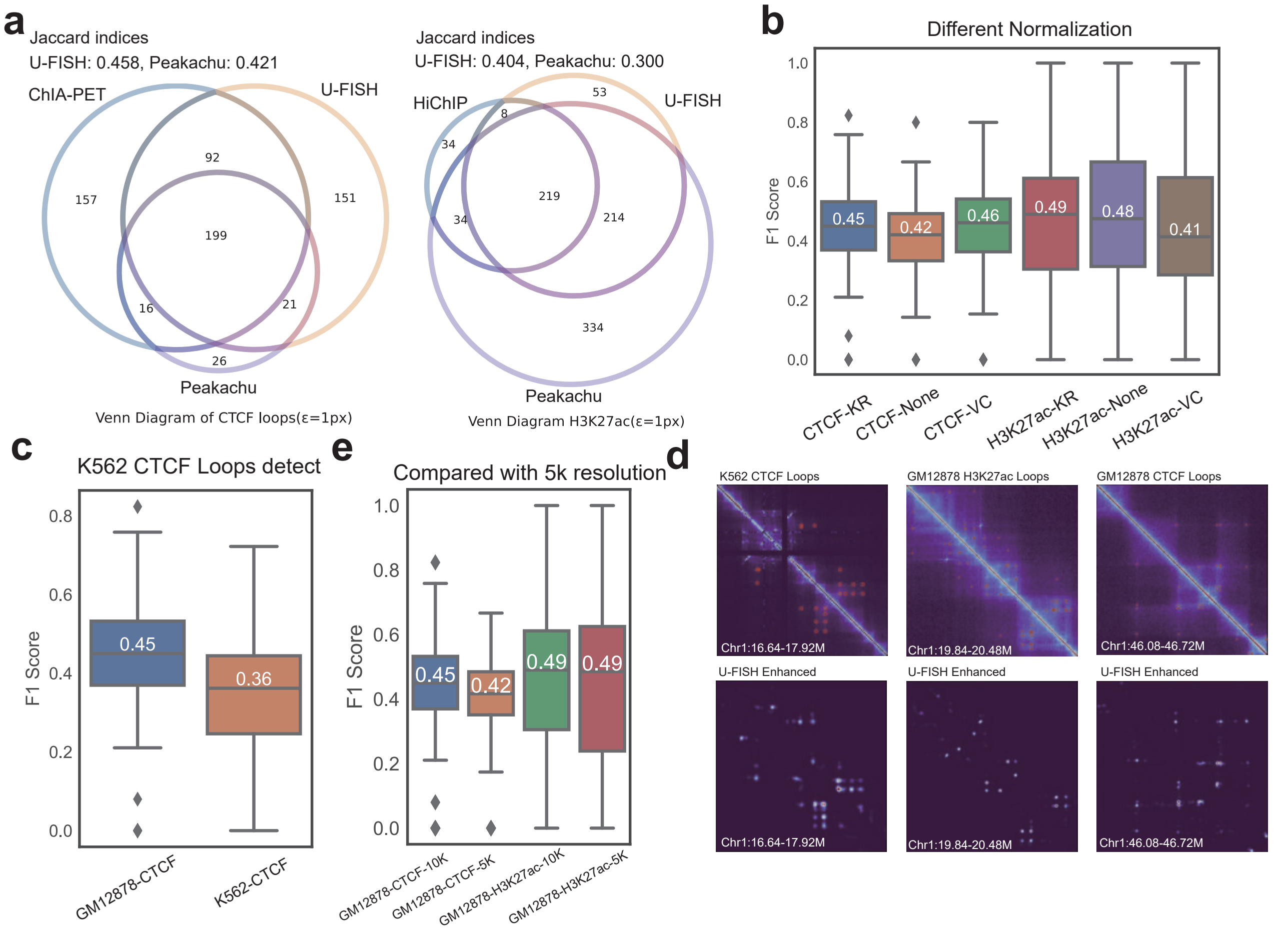

Fig. S14: Supplementary experimental results on Hi-C loop detection. **a,** Venn diagrams and Jaccard indices between the ground truth and the prediction results of U-FISH and Peakachu on the two loop datasets (ChIA-PET and HiChIP). **b,** Performance comparison of U-FISH loop prediction under different Hi-C matrix normalization/balancing methods. **c,** The predictive performance on K562 data of the model trained using the GM12878 matrix and CTCF loops. **d,** The enhancement effect of the U-FISH model trained on the GM12878 dataset on both the GM12878 matrix and the K562 matrix. **e,** The predictive performance of U-FISH on the 5k resolution Hi-C interaction matrix (GM12878), as well as a comparison with the predictive performance at 10k resolution.
