# Supplementary material for: U-FISH: a fluorescent spot detector for imaging-based spatial-omics analysis and AI-assisted FISH diagnosis
Source: Genome Biol. 2025 Sep 1;26:261. doi: 10.1186/s13059-025-03736-x (PMC12400573; doi:10.1186/s13059-025-03736-x)
Supplement: Supplementary file 2 — Additional file 2: Supplementary Tables. [file 13059_2025_3736_MOESM2_ESM.pdf]

Supplementary Tables

Ablation Setting	Current setting	Replacement	F1 (%)	delta (%)
Original			89.42 ± 0.79	-
Down sampling	3x3 Conv	MaxPool	89.41 ± 0.35	-0.01
Up sampling	Nearest neighbor	Transposed convolution	88.88 ± 0.20	-0.54
Residual connections	Yes	Conv Module	87.44 ± 1.25	-1.98
CBAM	Yes	Residual Conv Module	87.35 ± 1.08	-2.07
Batch size	16	8	89.31 ± 0.05	-0.11
	16	32	87.60 ± 0.57	-1.82
Optimizer	Adam	SGD	74.68 ± 5.16	-14.74
	Adam	RMSProp	87.31 ± 0.12	-2.12
Loss	DiceRMSELoss	RMSELoss	88.34 ± 0.64	-1.08
	DiceRMSELoss	DiceLoss	88.78 ± 0.19	-0.64
Target image	Gaussian	Dialate	88.03 ± 0.28	-1.39
	Gaussian	Single Pixel	84.44 ± 1.74	-4.98

Table. S1: Comparison of the impact of model configuration on mean F1 score (in percent)

Name	# of Patches(512*512)	Simulated(Y/N)	# of spots
MiP-Seq(RCA)	1052	N	465915
MERFISH	550	N	25286
smFISH(DeepBlink)	633	N	396663
ExSeq	480	N	20430
seqFISH	451	N	112519
simfish	500	Y	515000
DeepSpot	500	Y	99242
**Total**	**4166**		**1635055**

Table. S2: Dataset Sources and Statistics
